# Clustering based approach for population level identification of condition-associated T-cell receptor β-chain CDR3 sequences

**DOI:** 10.1186/s12859-021-04087-7

**Published:** 2021-03-25

**Authors:** Dawit A. Yohannes, Katri Kaukinen, Kalle Kurppa, Päivi Saavalainen, Dario Greco

**Affiliations:** 1 Research Programs Unit, Translational Immunology, University of Helsinki, Helsinki, Finland; 2 Department of Medical and Clinical Genetics, University of Helsinki, Helsinki, Finland; 3 Department of Internal Medicine, Faculty of Medicine and Health Technology, Tampere University Hospital, Tampere University, Tampere, Finland; 4 Department of Pediatrics, Tampere University Hospital and Center for Child Health Research, Tampere University, Tampere, Finland; 5 Faculty of Medicine and Health Technology, Tampere University, Tampere, Finland; 6 BioMediTech Institute, Tampere University, Tampere, Finland; 7 Institute of Biotechnology, University of Helsinki, Helsinki, Finland

**Keywords:** TCR differential abudance analysis, Celiac disease associated TCR clonotypes, Immune repertoire analysis, TCR repertoire analysis, Immuno-informatics, Antigen-specific TCR identification, Computational antigen-specificity identification, TCR clustering

## Abstract

**Background:**

Deep immune receptor sequencing, RepSeq, provides unprecedented opportunities for identifying and studying condition-associated T-cell clonotypes, represented by T-cell receptor (TCR) CDR3 sequences. However, due to the immense diversity of the immune repertoire, identification of condition relevant TCR CDR3s from total repertoires has mostly been limited to either “public” CDR3 sequences or to comparisons of CDR3 frequencies observed in a single individual. A methodology for the identification of condition-associated TCR CDR3s by direct population level comparison of RepSeq samples is currently lacking.

**Results:**

We present a method for direct population level comparison of RepSeq samples using immune repertoire sub-units (or sub-repertoires) that are shared across individuals. The method first performs unsupervised clustering of CDR3s within each sample. It then finds matching clusters across samples, called immune sub-repertoires, and performs statistical differential abundance testing at the level of the identified sub-repertoires. It finally ranks CDR3s in differentially abundant sub-repertoires for relevance to the condition. We applied the method on total TCR CDR3β RepSeq datasets of celiac disease patients, as well as on public datasets of yellow fever vaccination. The method successfully identified celiac disease associated CDR3β sequences, as evidenced by considerable agreement of TRBV-gene and positional amino acid usage patterns in the detected CDR3β sequences with previously known CDR3βs specific to gluten in celiac disease. It also successfully recovered significantly high numbers of previously known CDR3β sequences relevant to each condition than would be expected by chance.

**Conclusion:**

We conclude that immune sub-repertoires of similar immuno-genomic features shared across unrelated individuals can serve as viable units of immune repertoire comparison, serving as proxy for identification of condition-associated CDR3s.

**Supplementary Information:**

The online version contains supplementary material available at 10.1186/s12859-021-04087-7.

## Background

Targeted high-throughput sequencing of T-cell receptors, RepSeq, has enabled in-depth profiling of immune repertoires [1]. One critical application of RepSeq technology is the identification of condition-associated T-cell clones based on observed changes in T-cell clone frequencies. This allows the tracking of immune cells that have expanded or contracted following antigen exposure or treatment. Such analysis, however, is complicated by the fact that T-cell receptor (TCR) sequences are highly diverse, with estimated tens of millions of unique TCR expressing T-cell clones largely unique to individuals [2, 3], making direct comparison of T-cell clone abundances across multiple sample groups challenging.

A frequently used approach to the identification of condition-specific clonotypes across sample groups is the investigation of the so called public clonotypes (represented typically by unique TCR CDR3 sequences), which are commonly observed across many individuals [4–11]. However, such shared clonotypes make up a small portion of the total immune response in each individual. For instance, others and we have found that only around 10% of the response to gluten in celiac disease (CD) patients involves public CDR3 sequences [9, 12]. Thus, more should be learned about the adaptive immune response by also studying the private response. There are currently few methods that allow detection of both private and public disease-relevant clonotypes. DeWitt et al*.* reported a method that compares frequencies of clonotypes in repertoires sampled from the same individual to identify differentially abundant clonotypes, thus identifying disease-relevant clonotypes within each individual [13]. An improved variant of the method in DeWitt et al*.* accounting for time-dependent variation was also reported [14]*.* A recent method called ALICE [15] allows detection of relevant clonotypes from single repertoires by comparing observed number of TCR neighbors against expected number of neighbors estimated from data generated using a statistical TCR recombination model. Pogorelyy et al*.* also reported a Bayesian statistical method for comparing and detecting expanded/relevant clonotypes between repertoires of same individual at different time points [16]. These methods allow the identification of interesting clones, which are also private to individuals, but do not allow the direct investigation of differentially abundant clonotypes at the population level although ad hoc combining of results from multiple samples is still possible. Moreover, except ALICE, the methods require acquisition of multiple samples from each individual. Thus, there is currently a need for methods that perform direct population level comparison of clonal differential abundance for the identification of condition-specific T-cell clonotypes in longitudinal or case–control RepSeq datasets.

We recently showed that over-represented amino acid motifs in CD-associated TCR CDR3β sequences, originally identified from tetramer binding antigen-reactive T-cells [17, 18], were also detectable from the unsorted total peripheral blood immune repertoires of celiac disease patients despite the immense repertoire diversity [9]. This observation and closer inspection of the CD-associated TCR CDR3β sequences, strongly suggested that CDR3β sequences associated to CD exhibit sequence-level similarities that can be used to group them into clusters, reflecting similar immuno-genomic features involved in the immune reaction that are possibly shared by patients. Such high sequence similarity had been observed in B-cell receptors (BCRs) associated to chronic lymphocytic leukemia (referred to as BCR stereotypy), with potential clinical relevance [19, 20]. More recently, Dash et al*.* and Glanville et al*.* have shown that antigen-specific TCR sequences collected from different patients could be clustered into antigen specificity groups that share sequence similarity [21, 22]. Overall, recent RepSeq studies have reported sequence similarity in CDR3 sequences associated with conditions, a characteristic of the immune response that could be harnessed for the identification of condition relevant CDR3 sequences from total unsorted repertoires by comparing sample groups.

In this work, we propose differential abundance analysis at the level of shared clusters of T-cell receptor CDR3β sequences, called sub-repertoires, to enable identification of disease relevant clusters of CDR3β sequences by comparing RepSeq experimental groups. We first applied within-sample CDR3β clustering to reduce the diversity of immune repertoires into manageable and comparable units of analysis. We showed that these clusters of receptors, made up of clonotypes with highly similar frequencies of nucleotide or amino acid subsequences (k-mers), form biologically meaningful units of analysis since they are commonly present in repertoires of unrelated individuals. We then performed statistical differential abundance analysis at the level of these sub-repertoires for the identification of condition-specific CDR3β clonotypes. We also showed that this methodology allows successful detection of condition-associated CDR3 sequences, both private and public, from immune repertoire datasets of unrelated HLA-matched celiac disease patients, and yellow fever virus vaccination volunteers, by comparing groups of samples at the population level.

## Results

**Fig. 1:**
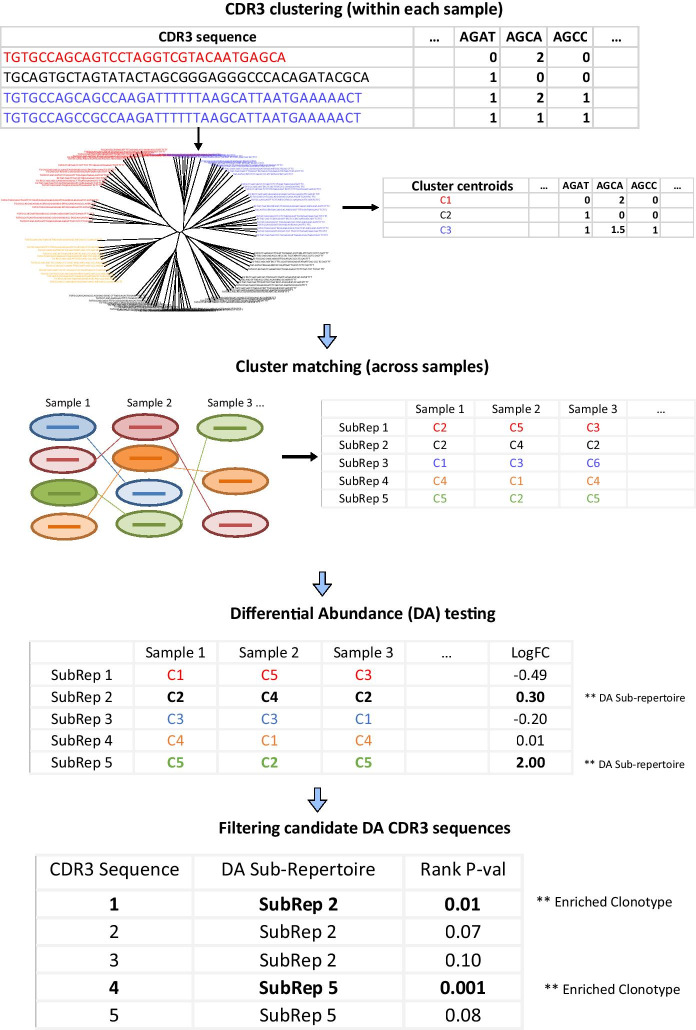
Schematic of the clustering-based differential abundance detection methodology for CDR3 repertoire HTS datasets

We hypothesized that clustering of CDR3s in the global repertoire not only reduces the enormous diversity of the immune repertoires into manageable units, but also has the potential for allowing indirect detection of condition associated CDR3s by first comparing the abundance of CDR3 clusters between sample groups (Fig. 1). To investigate the validity of such an approach, we first evaluated if a cluster of CDR3β sequences in one sample could be similar, in terms of subsequence composition, to another cluster in another sample. Importantly, the cluster must be closer to its match in another sample than it is to other clusters of CDR3βs in its home sample, ideally incorporating information not just from the germline-encoded variable (V), diversity (D) and/or joining (J) regions but also from the non-templated nucleotides in the N1 and N2 regions, signifying conserved immuno-genomic as well as antigen induced TCR selection features across samples.

**Fig. 2:**
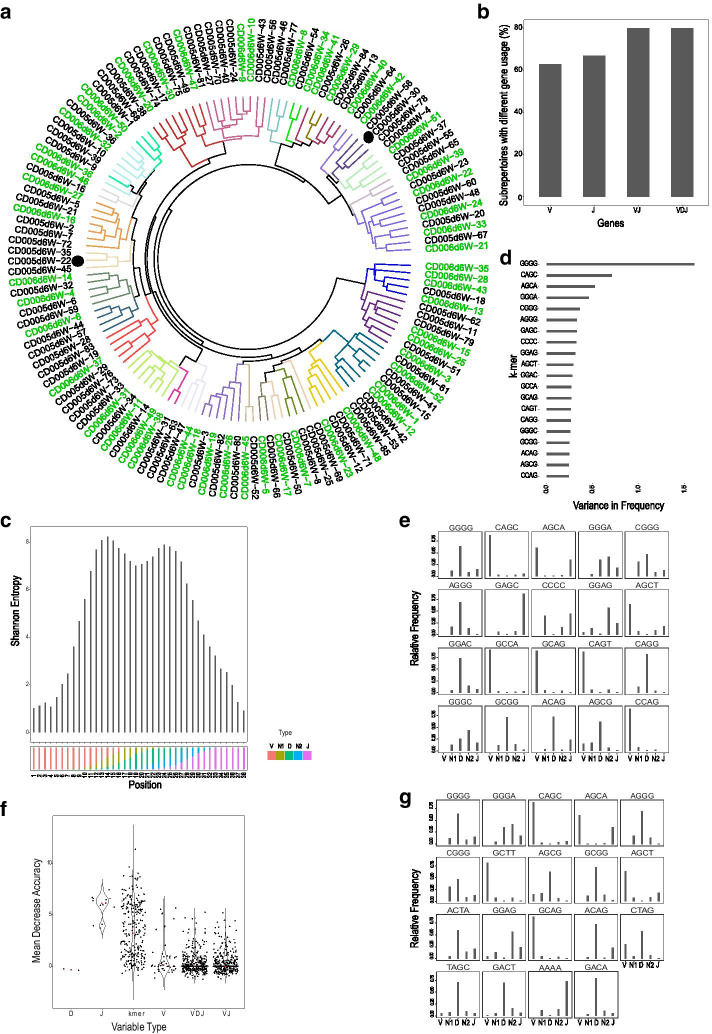
CDR3 sub-repertoire matching in samples of two unrelated individuals. **a** hierarchical clustering of CDR3 cluster centroids from samples CD005 (black) and CD006 (green) from our CD PBMC dataset identified 32 sub-repertoires of which 30 (94%) had cluster representatives from both samples. Branch colors indicate sub-repertoires. Only 2 of the 32 (6%) sub-repertoires (shown in black dots) are homogenous, containing cluster centroids from only one sample. **b** V-, J-, VJ- and VDJ gene usage frequency was compared between clusters coming from the two samples, the percentage of sub-repertoires with significantly different gene usage with p value below 0.05 (using chi-square test of independence) is shown. **c** Number of different possible 4-mers that start at each position is estimated using Shannon’s entropy for 42nt long CDR3s, highest entropy is observed in positions in which CDR3s have the N1 and N2 region. Similar result was obtained in all samples. 4-mers that are not completely within the N1 or N2 region but either end or start in the regions are counted towards them. **d** Top 20 4-mers with the highest variance in frequency across the 5000 subsampled CDR3s within a single sample (CD005) is shown. **e** The frequency of where (in V, N1, D, N2, J) the top 20 most variable 4-mers are found in the CDR3s is shown. **f** The classification importance of k-mers and genes in distinguishing 4-mer based clusters within a single sample (CD005) is shown. **g** The frequency of where (in V, N1, D,N2, J) the top 20 most discriminative 4-mers (ordered left to right) are found in the CD005 repertoire is shown

We observed that such clusters of CDR3βs, with closely similar subsequence composition, exist across samples in unrelated individuals. For example, for the two CD PBMC repertoire samples CD005 and CD006 (unrelated celiac disease patients), we first subsampled 5000 CDR3βs each from their total unique nucleotide CDR3β sequences, and performed unsupervised clustering of the CDR3βs within each sample. The centroids of all clusters from both samples were then pooled, and clustered again to identify matching CDR3β cluster centroids (steps 1 and 2 on Fig. 1). Out of the 32 identified centroid clusters i.e., sub-repertoires, 30 (~ 94%) had centroids representing CDR3β clusters from both samples (Fig. 2a). The same analysis on all 8 samples of CD PBMC dataset showed similar result, with all sub-repertoires having representative centroids from multiple samples (Additional file 1: Fig. 2Sa). As this could be expected if clusters are largely defined by germline-encoded V, D and J genes, we performed a number of analyses to assess the extent to which such nucleotide 4-mer defined clusters are influenced by germline sequences. We first compared V-gene, VJ-gene, VDJ-gene and J-gene usage profiles between matching clusters in sub-repertoires. In both the two sample and all 8 sample analyses, germline genes do not completely explain the subsequence composition similarity in matching clusters, with around 50% for V-gene and J-gene, and up to 80% for VJ- and VDJ- genes, of the sub-repertoires containing matching clusters with significantly different gene usage profiles (Fig. 2b and Additional file 1: Fig. 2Sb and c). We then looked at the diversity of nucleotide 4-mers that start at each position of CDR3s (from a single sample) starting from the conserved cysteine to the end of the CDR3s. This was done for CDR3s with the most prevalent CDR3 length of 42 (Additional file 1: Fig. 2Sd). As we expected, we generally observed low diversity/variation of possible 4-mers in positions that only have germline genes and the highest 4-mer diversity in positions that have N1 and N2 regions (Fig. 2c), suggesting k-mer frequency estimates would be most different among k-mers that appear in these high entropy regions. We thus evaluated the regions of CDR3s from where 4-mers with the highest variation in frequency (among CDR3s) originate. Among the top 20 such high variation 4-mers, we observed that while some mainly originate from V or J-gene regions, many originate from the N1, D, N2 regions including the k-mer with the highest variance (GGGG) which originates primarily from the N1, D, N2 regions (Fig. 2d, e), suggesting that k-mers with the most potential for discriminating between CDR3s arise from such high diversity regions. To confirm this further, we built a classification model using Random Forests with clusters (within sample clusters) or sub-repertoires (across sample cluster matches) as classes, and nucleotide 4-mers, one hot encoded V-genes, J-genes, VDJ, and VJ as variables, and evaluated the importance of the variables in classifying clonotypes into clusters or sub-repertoires. This showed, although J-genes have a higher median importance, k-mers in general have the highest importance in defining the classes (in clusters within a sample, Fig. 2f), with more k-mers having importance values above the highest valued J-gene than the number of all possible 13 J-genes (in the 8 sample sub-repertoires, Additional file 1: Fig. 2Se). Analysis of where k-mers within the top 20 most discriminative variables originate showed more than half arise primarily from N1,D,N2 regions both for clusters within sample (Fig. 2g) and even more so in the sub-repertoires across the 8 samples (Additional file 1: Fig. 2Sf), giving similar results to what we observed with the most variable k-mers. Similar analyses with all k-mers that primarily (most frequently) originate from any of the N1, D and N2 regions showed that a subset of such k-mers had the highest importance scores compared to all variables both for classifying within sample clusters or cross-sample sub-repertoires (Additional file 1: Fig. 2Sg and 2Sh). In summary, these results suggest that CDR3β clusters and sub-repertoires defined by k-mers capture discriminative information from the non-templated, junctional insertion/deletion regions and do not simply recapitulate clonotype grouping by simple germline gene usage, as a result leading to better clustering of clonotypes with shared immunological information both within and across samples.

**Fig. 3:**
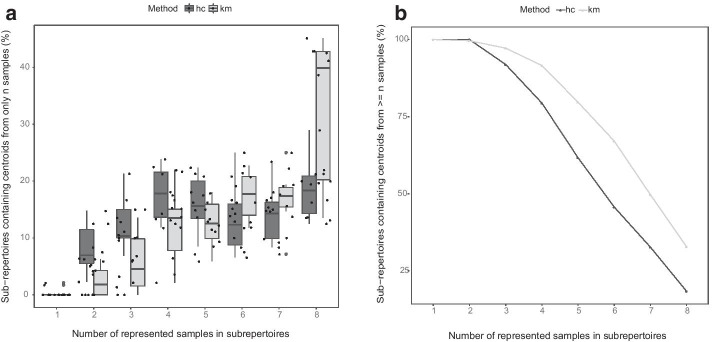
Sub-repertoire detection across many samples. CDR3 clustering, and sub-repertoire detection using both hierarchical clustering (hc) and k-means (km) clustering of the CDR3 cluster centroids was performed for all 8 CD PBMC samples. **a** Shows proportions of sub-repertoires containing CDR3 cluster centroids from only n samples, dots are the estimate from each of 10 analyses from subsampled repertoires with sequencing depths of 1 to 10 thousand unique nucleotide CDR3s per sample. **b** The cumulative proportion of sub-repertoires containing representative clusters from n samples or more is shown; the cumulative at each n is computed as the mean proportion of n represented samples from the 10 resample analyses

We then investigated the extent of sub-repertoire sharing when there are more number of samples in the analysis. We performed the clustering and cluster matching analysis on all 8 samples of our celiac disease (CD) PBMC datasets, from both pre-gluten challenge (day 0) and post-gluten challenge (day 6) conditions, at different sequencing depths per sample (Fig. 3). The proportion of sub-repertoires containing representative centroids from only one sample was negligibly low in the ten analyses done at different repertoire depths (Fig. 3a), while at minimum 10–20% of sub-repertoires contained CDR3 cluster centroids from all 8 samples. But cumulatively, more than ~ 40% (with hc matching) and 60% (with km matching) of the sub-repertoires contain centroids from at least 6 of the 8 samples (Fig. 3b), suggesting that enough of the sub-repertoires are present in multiple samples to allow comparison of sub-repertoire abundance at the population level, and to enable indirect detection of condition relevant CDR3s.

**Fig. 4:**
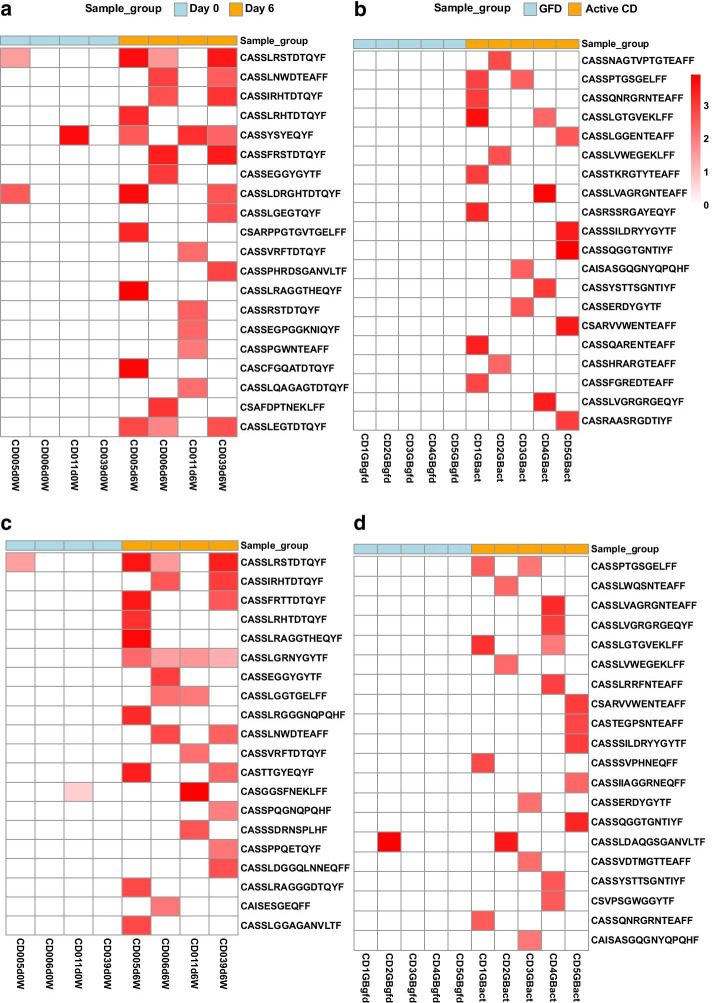
Differentially abundant CDR3β sequences identified by the method. The top 20 significantly differentially enriched CDR3β sequences during gluten exposure are shown for **a** CD PBMC and **b** CD Gut datasets when using nt 4-mer feature vectors. The result obtained using aa 3-mer feature vectors is on **c** for CD PBMC and **d** for CD Gut datasets. Abundance is shown in log10 scale, from low abundance (white) to higher abundance (red). GFD treated samples are shown in light blue and gluten exposed samples are shown in orange bars at the top

Applying the method on our datasets, for the CD PBMC dataset (n = 4), the method identified 2315 and 2467 CDR3β sequences that showed significant enrichment following gluten exposure when using nucleotide 4-mer and amino-acid 3-mer feature vectors respectively. For the CD Gut dataset (n = 5), the method identified 2291 and 2404 enriched CDR3βs during active celiac disease when using nucleotide 4-mer and amino-acid 3-mer feature vectors respectively. Figure 4 shows the top 20 enriched CDR3βs detected from both datasets when using nucleotide 4-mer and amino-acid 3-mer feature vectors (for the list of all detected CDR3s see Additional file 2). Considering the high diversity of immune repertoire datasets, the results of the analyses performed with nt 4-mers and aa 3-mers showed high overlap (as high as 70% in CD PBMC and 78% in CD Gut, Additional file 1: Figure 3Sa and b), suggesting results of comparable relevance could be obtained by either feature vectors, although the latter is more computationally expensive.

To assess the method’s sensitivity to detect CD associated clonotypes, and its ability to detect enrichment beyond clonotype size differences due to sampling variation, we applied the nucleotide 4-mer analysis on same condition CD PBMC samples prepared by a pooling and random sampling strategy (see the *Analysis of same condition samples* section in Additional file 1). For both pre-gluten exposure day 0 and post-gluten exposure day 6, ten unpaired comparisons of same condition, randomly drawn samples, identified negligibly low numbers of enriched clonotypes with a mean of 1.5 among day 0, and 2.4 among day 6 same condition comparisons (Additional file 1: Fig. 5S), suggesting that the method is sensitive to sampling variation in picking relevant clonotypes.

**Fig. 5:**
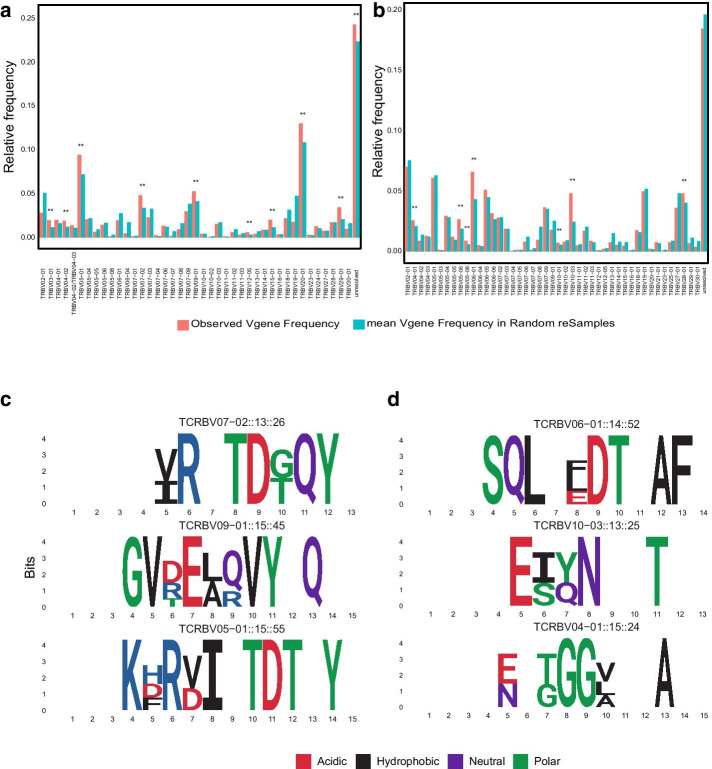
Characteristics of the differentially abundant CDR3β sequences in CD PBMC and CD Gut. The differentially enriched CDR3β sequences had biased usage of TRBV genes that are known to be over-represented in gluten reactive CDR3β sequences in previous studies, such as TRBV07-02 and TRBV09-01 from CD PBMC (**a**), and TRBV06-01 from CD Gut (**b**) (observed frequencies are shown in red, mean frequency from randomly generated sets of CDR3s are shown in blue). Significantly over-used amino acids at each position are shown for the enriched CDR3β sequences that use TRBV genes detected to be over-used from CD PBMC (**c**) and CD Gut (**d**), amino acids are colored according to their properties. The information content of significantly overused amino acids at each position is shown in bits on the y-axis. TRBV and per-position amino acid over-usage is assessed by comparing the observed frequencies in the set of differentially enriched CDR3s to that obtained by chance in 100 randomly sampled CDR3s of same size, TRBV gene and CDR3 length, with p < 0.05 considered significant (gene names indicate TRBVgene::CDR3 length::number of CDR3s in the enriched list with the Vgene and CDR3 length). The results from using nt 4-mer feature vectors are shown

For method validation, we compared the detected CD associated enriched CDR3βs to known celiac disease associated CDR3β sequences in the literature. The detected enriched CDR3βs had significantly increased usage of previously reported TRBV-genes associated with gluten-reactive CDR3β sequences [17, 18, 23–26], with the enriched CDR3βs from CD PBMC dataset showing biased usage of TRBV-gene families 4,5,7, and 9, specifically TRBV04-02, TRBV07-02 and TRBV09-01 (Fig. 5a, and Additional file 1: Fig. 4Sa). The enriched CDR3βs from CD gut dataset also showed biased usage of previously reported TRBV06-01 in addition to new interesting genes such as TRBV10-03 (Fig. 5b, and Additional file 1: Fig. 4Sb). Furthermore, per-position amino acid usage analysis of the enriched CDR3s bearing some of the over-used TRBV genes provides interesting insights. Besides detecting CDR3βs with the already known amino acid motifs in gluten-reactive CDR3βs with a dominant usage of Arginine (R) in position 6 [17, 18] (Fig. 5c and Additional file 1: Fig. 4Sc), the method identified other previously unreported over used genes such as TRBV03-01,TRBV15-01 and TRBV10-01 in the detected enriched CDR3s with previously under-appreciated per-position amino acid usage patterns, such as the previously reported (although in Humanized HLA-DQ8 transgenic mice) over-usage of Glutamic acid (E) and Aspartic acid (D) at position 6 of gluten specific TCR CDR3βs [27] (Additional file 1: Fig. 4Sc and d). In addition, except in the nucleotide subsequence based analysis of CD Gut, the list of enriched CDR3βs from both CD PBMC and CD Gut contained significantly high number of previously reported CD-associated CDR3β sequences by Qiao et al*.*, Han et al*.* and Petersen et al*.* [17, 25, 26] than was expected by chance, as determined by both using a randomization test or a straight forward comparison to the proportion of previously known celiac disease associated CDR3s in the total repertoire of the combined dataset of all samples (Table 1 and Additional file 1: Table 4S, see Additional file 1: Tables 2S and 3S for the list of previously known celiac disease associated CDR3 identified by the method). There was also high overlap between the known CD clonotypes detected by the nt 4-mer and aa 3-mer approaches (Additional file 1: Fig. 3Sc and d). The method detected known CD associated CDR3s mostly from CD4 + T-cells in the CD PBMC (as determined by referring to the T-cell types in the previous reports bearing the CDR3s) and CD8+ T-cells from the CD gut datasets (Table 1). There was no detection of any known CD associated CDR3βs among the list of de-enriched sequences obtained from both nucleotide 4-mer and amino-acid 3-mer based analyses of CD PBMC, while for CD Gut, two known CD clonotypes were de-enriched in the nucleotide 4-mer and one in the amino-acid 3-mer analyses (Additional file 1: Table 3S).

**Table 1:** Previously known condition-associated CDR3s in the list of DA enriched CDR3s identified by the method (using randomization test)

Dataset	known condition-associated CDR3s in all samples (*likely CD4, CD8)	Feature space	Known condition-associated CDR3s in DA enriched (likely CD4, CD8)	Permutation test p value (CD4, CD8)
CD PBMC	56 (20, 36)	nt 4-mer	14 (9, 3)	p = 0.0 (0.0, 0.09)
CD PBMC	56 (20, 36)	aa 3-mer	10 (7, 2)	p = 0.0 (0.0, 0.33)
CD GUT	50 (23, 24)	nt 4-mer	3 (0, 3)	p = 0.48 (1, 0.05)
CD GUT	50 (23, 24)	aa 3-mer	5 (1, 4)	p = 0.13 (0.85, 0.02)
YFV PBMC	12,092	nt 4-mer	697	p = 0.0
Twin YFV PBMC	5730	nt 4-mer	2058	p = 0.0

^*^Previously reported CD associated CDR3s are assumed likely CD4 and CD8 based on their reported CD4 and CD8 status in the papers they were reported. For some such information was not available

*p* values show non-significant results

We also used the method on the publicly available yellow fever vaccination T-cell CDR3β repertoire datasets, YFV PBMC (n = 9) [13] and YFV PBMC from twins (n = 6) [16] to identify CDR3β sequences responding to the YF-17D vaccine. We compared the total pre-vaccination (day 0) PBMC repertoires to the total post-vaccination (day 14 or day 15) PBMC repertoires. The list of enriched CDR3βs the method identified contained significantly high numbers of vaccine induced CDR3βs that were reported in the original publications (Table 1 and Additional file 1: Table 4S. For the list of all detected YFV associated CDR3s see Additional file 2). For the YFV PBMC dataset, it identified 2620 enriched clonotypes across all individuals of which 697 (~ 27%) are in the YFV-induced day 14 effector CD8+ T-Cell CDR3βs that were statistically determined to be expanded (compared to day 0 total repertoires) in the original publication. Similar analysis in the original DeWitt et al. study identified 848 such enriched clonotypes across all samples that were also present in the expanded YFV-induced day 14 effector CD8+ T-Cell CDR3βs, showing that we obtained comparable results by comparing the sample groups directly. Since we compared the total day 14 versus day 0 repertoires, the identified enriched clonotypes contain not just CD8+ but also expanded T-cells that are CD4 + and other CD8+ T-cells that do not have the exact markers used to sort for the YFV-induced day 14 effector CD8+ T-Cell population, as was noted similarly in the original publication [13]. For the twin YFV PBMC dataset, the method identified 4152 enriched clonotypes of which 2058 (~ 50%) were present in the expanded clonotypes reported by Pogorelyy et al*.* [16]. Interestingly, 223 of the detected enriched clonotypes for the twin YFV PBMC dataset were present in the expanded YFV-induced day 14 effector CD8+ T-Cell CDR3βs of the YFV PBMC dataset from DeWitt et al*.*, of these, 122 were detected only by our method and were not present in the expanded clonotypes reported by the twin YFV PBMC study by Pogorelyy et al*.* [16]. Furthermore, we checked the number of YFV (A02-NS4b_214−222_–specific) versus CMV (cytomegalovirus) specific TCRβ sequences in our list of enriched clonotypes as was done in the original study by Pogorelyy et al*.* Our method identified no CMV-specific clonotypes, and identified significantly more published YFV-specific clonotypes with exact matches than was reported among the expanded clonotypes in the original study (Additional file 1: Table 5S). Overall, the results from the YFV datasets suggest that the method can find utility in various study types where detection of T-cell clonotypes with significant expansion is required.

To benchmark method performance, we compared the method against four recently published methods, as well as against results obtained by the method when using germline gene usage for grouping clonotypes instead of k-mer based clustering (in step 1 and 2 of the method). Using our CD PBMC dataset, we looked at how many of the 56 previously known CD-associated CDR3s (that exist in the CD PBMC dataset) the methods detect, as well as evaluated the methods’ recall and precision. Our method (RepAn, nt 4-mer based) identified 14 of the 56 known CD associated CDR3s as differentially enriched, whose proportion in comparison to total identified enriched clonotypes is similar to all other methods except ALICE and the public only methods, which are all highly conservative and thus have much lower recall (Additional file 1: Table 6S). The method has the second highest recall and precision, next to the DeWitt’s method or third highest in precision if we include the Yohannes et.al method that detects only public clonotypes. It outperformed all germline gene usage based variants of RepAn in both recall and precision. Thus, the method provides detection of both public and private clonotypes with comparatively high recall and precision, while being the only method that allows direct population level analysis (Additional file 1: Table 6S, see Additional file 2 for the list of the 56 CD-associated CDR3s in the CDPBMC dataset along with those detected by the methods). We note that the shared detection between any two of these methods is rather low (Additional file 1: Fig. 6S) and thus a criteria of detection in at least one other method for assessing precision and recall was used.

## Discussion

The computational pipeline we present in this work allows comparison of total immune repertoires between sample groups to identify both public and private CDR3 clonotypes associated with conditions. To our knowledge, this is the only currently available method for direct population level comparison of TCR CDR3 repertoires, with incorporated population-wide statistical assessment of TCR clonotypes’ condition-relevance, and thus provides improved ability for examining and monitoring immune responses.

We made two main assumptions in our proposed method. Firstly, we assumed that the immune repertoire specific to an antigen or epitope would contain T-cell clones with high similarity in their T-cell receptors (TCRs) forming a cluster (or group of clusters) that is distinct from other T-cells not specific to the antigen. This assumption originally stemmed from observations of the celiac disease associated CDR3β sequences in our and other previous studies [9, 17, 28]. Recent works by Dash et.al. and Glanville et.al. [21, 22] showed that tetramer sorted antigen specific TCRs from different individuals have high sequence similarity and could be grouped into clusters with common specificity to an antigen, further justifying the validity of our assumption. Secondly, we assumed that the clusters of TCRs specific to an antigen encode an important immuno-genomic information in the immune response that is shared across unrelated individuals and could probably be detected from the global repertoire and across treatment conditions. We show that k-mer frequency vectors capture immune information from all parts of CDR3 sequences beyond what could be captured by simple grouping of CDR3s based on germline gene usage, with the most discriminative k-mers between sub-repertoires dominated predominantly by those arising also from non-templated insertion/deletion regions of CDR3s. Our method based on such nucleotide 4-mers or amino acid 3-mers allowed dissection of total immune repertoires into units (meaningful sub-repertoires) that exist across individuals, and thus successfully identified previously reported and new condition-associated CDR3β sequences (both private and public) from the datasets we analyzed with better precision, demonstrating the validity of our assumption.

Various ways of representing the CDR3 sequences have been used in recent immune repertoire studies in order to ascertain sequence similarity. Thomas et al. used Atchley factors to represent amino-acid subsequences of CDR3 [29, 30], although applied for classification of total repertoire samples. Direct comparison of the receptor sequences is also possible without CDR3 representation by numeric vectors. Dash et al. defined a metric called TCRdist, that uses the amino acid receptor sequences directly, with a weighted Hamming distance of the amino acid sequences of not only the CDR3, but also the CDR1 and CDR2 of both alpha and beta chains to determine the distance between two T-cell receptors [21], while Glanville et al. combined hamming distance between amino acid CDR3 sequences, with usage patterns of k-mer subsequences in structurally determined positions of high antigen contact propensity to measure distances between pairs of CDR3s [22]. We represented CDR3 sequences using a simple, high dimensional subsequence frequency vector, which was then used to define distance in that feature space and cluster CDR3 sequences into similar groups. Greiff et al. recently found such immuno-genomic representation of CDR3s to be highly meaningful in allowing the prediction of private versus public CDR3 sequences with high accuracy [31]. We showed that such k-mer frequency based representation allows detection of both public and private condition relevant clonotypes. Particularly for the private clonotypes, their importance is assessed by proxy, i.e., via the detection of their sequence composition similarity, and importantly their repeated detection as relevant clonotypes within sub-repertoires deemed differentially abundant in multiple downsampled analyses, providing replicated evidence of relevance.

We evaluated method performance by comparing the detected CDR3s to known antigen binding condition-associated CDR3s from previous studies. The method detected statistically significant numbers of known condition-associated CDR3s (for both celiac disease and yellow fever vaccination datasets) than could be obtained by chance. The detected CDR3s showed significant bias in V-gene and per-position amino acid usages typical of known condition-associated CDR3s, validating the high usability of the proposed method. The method also compared favorably to recently published methods in detecting known celiac disease associated clonotypes enriched in our dataset. It showed relatively high recall and precision in an assessment using concordant detection of enriched clonotypes between the methods to define possible true and false detection.

While we did not directly assess the impact of HLA-type differences in the method’s performance, we presume that the method could pick HLA-associated TCRs unless HLA-types are fairly randomized in the compared groups. Our CD datasets were matched for at least one copy of HLA-DQ2, which has the strongest genetic association to CD. No HLA information was available for the public dataset YFV PBMC, while the twin YFV PBMC dataset was HLA-matched for HLA-A*02, which restricts an immunodominant YFV epitope (NS4b_214−222_), but is not otherwise the only HLA association to YFV [32, 33], thus the results from the YFV datasets were largely HLA-independent. The method can be extended to include k-mer frequencies in the other complementarity-determining regions of the TCR beta-chain (CDR1 and CDR2), and alpha-chain (CDR1, CDR2 and CDR3), capturing a more thorough information of clonotypes’ specificity to antigens. In the case of CDR1 and CDR2 (encoded by the V genes), this could allow the method to be more sensitive to the effects of HLA, as CDR1 and CDR2, unlike CDR3, make contact only with the Major histocompatibility complex (MHC), and not directly to the antigen peptide in the pMHC site, modulating TCR specificity indirectly [34].

Condition-associated TCR CDR3s are not fully known for many multifactorial autoimmune or cancer related diseases. For diseases with known antigen, such as celiac disease (CD) where some information about the antigen (gluten) is known, specific protocols like tetramers and/or sorting would have to be designed to characterize the antigen-specific T-cells, their CDR3s, and other phenotypes in detail. Although highly useful, such methods designed to select antigen-specific repertoires are unable to detect T-cell clones responding to other important immune targets other than gluten, either self or foreign, possibly ignoring a crucial part of the immune response that would explain the pathology all the more. Methods such as the one presented in this study that attempt to detect condition-relevant T-cell clones from total repertoires, without necessarily having prior knowledge of the immune target (or targets), coupled with techniques that profile the overall gene expression of the condition-associated T-cell clones, would potentially provide a highly comprehensive picture of the adaptive immune response. This leads to a much more complete understanding of such diseases in-terms of unraveling the hidden pieces of the puzzle, and could provide ways for the prediction of the unknown immune targets. For diseases in which the antigens are totally unknown, the application of such methods could lead to the identification of the associated CDR3 sequences that could serve as immune-response bio-markers with possible clinical application, as well as enabling their comparison to other known disease associated CDR3 sequences available in CDR3 databases [35, 36], potentially allowing prediction of their possible target antigen via specificity estimates that can be obtained using recently reported TCR-epitope specificity prediction methods [37].

Main limitations of the methodology include restricted repertoire representativeness and computational intensiveness, both arising from the immense diversity of immune repertoires. There are millions of unique CDR3 sequences in every person, each representing a T-cell clone, and most found only in a single person. Only tens to hundreds of thousands of unique CDR3s are being sampled with the current Repseq technology per sample. Since calculating pairwise distances for potentially hundreds of thousands of CDR3s is computationally intensive, if not infeasible, we adopted repeat resampling and applied the methodology a repeated number times with randomly selected smaller repertoire samples. The number of repeat resample runs chosen and the repertoire size of each repertoire resamples determines the computational resources required and exhaustiveness of the result. Additionally, usage of bigger k sizes for the k-mer frequency based CDR3 encoding is computationally intensive. While our choice of k = 4 for nt and k = 3 for aa is based on their successful applications in other studies (see Methods), analyses of ks from 2 to 8 for nt k-mers showed no substantial difference, or gain in detection capacity, while at the same time resulting in significant increase of computational requirements as k increases (Additional file 1: Fig. 7S). As Repseq datasets are already huge with thousands of clonotypes in multiple samples, the use of smaller k is a better tradeoff. We ran the method for all datasets on a supercomputer cluster using a single node with 24 cores. Running the method with 100 resample rounds for the CD PBMC and CD Gut datasets took around one hour using nt 4-mers, and 15 and 8 h respectively, when using aa 3-mers. A combined memory of approximately 100 GB was used for both nt and aa based analyses. For the two bigger YFV datasets, 600 resample rounds needed 40 to 50 h for an nt 4-mer based analysis with a maximum of 322 GB of memory used (Additional file 1: Table 7S). As the results from using nt 4-mers are highly comparable to those obtained by aa 3-mers, using the method with the nt 4-mer option is the better option for bigger datasets whenever clonotype nucleotide data is available.

## Conclusions

To conclude, by clustering CDR3 sequences into groups with similar immuno-genomic features, and finding their close matches across different samples, we showed that condition-associated CDR3 sequences that are private or public, and with significant differential abundance, can be detected by direct comparison of groups of samples. The approach paves the way for the identification of private or public CDR3s (and their features) associated with diseases or other important phenotypes such as HLA-type, further allowing comprehensive categorization and archiving of T-cell clonotypes. This has a vast potential in understanding the adaptive immune response in various disease conditions and disease development stages, identifying unknown self or foreign antigens in diseases with unknown immune targets, examining immunological history encoded in the immune repertoire, and possible early prediction of the adaptive immune response.

## Methods

Our methodology narrows down the highly diverse immune repertoire data and helps to identify disease associated differentially abundant CDR3 sequences by comparing samples from two treatment groups. As input, it expects high-throughput genomic T-cell receptor CDR3 sequences (of either CDR3α or CDR3β) in immunoseq format [38] for every sample, which is a tab-delimited text file with each row containing information about a CDR3 and its features (its Nucleotide & Amino acid sequences, CDR3 length, frequency, V-,D-,and J-gene segment usage, etc.). Additionally, it also requires information regarding the experimental condition of every sample. The method can also accept MiXCR [39] formatted cDNA repertoire data, in which case the analysis results should be interpreted as differential expression of clonotypes instead of clonal expansion (enrichment) or contraction (de-enrichment). Referring to each such repertoire file simply as a sample, the method processes the samples in four major steps to identify differentially abundant CDR3 sequences (Fig. 1):

1. CDR3 clustering: each CDR3 in a sample is first represented using a high dimensional k-mer frequency vector by counting the frequency of each possible contiguous nucleotide (nt) or amino acid (aa) subsequences in the CDR3. We have typically used k = 4 for nt k-mers, resulting in a feature vector size of 256, or k = 3 for aa k-mers resulting in a feature vector size of 8000. Our choices for these ks are based on previous successful applications of using sequence composition for meaningful unsupervised clustering in similarly high diversity datasets. Nucleotide 4-mer (tetranucleotide) frequencies have been used extensively in metagenomic binning for assignment of reads into taxonomic groups [40–42], and we surmised, its adoption in nucleotide composition based TCR grouping is reasonable as TCR repertoires may mirror the major groups of environmental antigens the TCRs engage. For amino acid TCRs, clustering a 3-mers based amino acid Atchley factor encoding of TCRs has been shown to allow better classification of a whole repertoire’s immunization status [29].
  Next, the high-dimensional k-mer frequency vectors are used to perform unsupervised clustering of the CDR3 sequences within each sample (using agglomerative hierarchical clustering, with the complete linkage method which showed better cluster stability with more number of clusters-or “zooming-in”, Additional file 1: Figure 1S). We employed the Euclidean distance to determine the distance between a pair of CDR3 k-mer frequency vectors. After the hierarchical clustering, the dynamic tree cut algorithm is used to define the CDR3 clusters in each sample [43].
2. Cluster matching across samples: For each cluster in each sample, the average frequency for each k-mer is computed from the members of the cluster to get the cluster centroid. Here, k-mer frequencies in member clonotypes are not weighted by CDR3 abundances in the underlying data in order to have centroid k-mer frequencies reflect only the basic subsequence compositional characteristics of clusters, and to avoid bias due to sequencing depth differences when matching across samples. The centroids of clusters from all samples are collected together, and unsupervised clustering of the centroids is performed using either hierarchical clustering or K-means to group centroids based on closeness in k-mer frequency profiles. When using k-means, clustering of the centroids is performed using a k that has the maximum optimal-k (oK) score:
  $${\text{oK}} = \left( {{\text{nS}} + {\text{avS}}} \right)/2,$$
  where nS is the proportion of clusters with silhouette value greater than the average silhouette (over all clusters), and avS is the average silhouette shifted to be between 0 and 1, by adding 1 and dividing by 2. oK values range from 0 to 1. To determine the k with maximum oK score, oK is computed for all k starting from the minimum to the maximum number of CDR3 clusters per sample observed across all samples (from the result of step 1).
  Next, each cluster of centroids is examined and, if multiple centroids from one sample are determined to be in the same centroid cluster, the clusters of such centroids are merged in the original sample and the centroid updated. Otherwise, all clusters of centroids representing matching clusters from multiple samples (not necessarily all samples) are retained.
  Given N samples, this step generates a cluster match table with oK rows and N columns, in which row entries represent CDR3 cluster labels from all samples that are close (or have matching) centroids, representing underlying features encoding conserved immunological features in all N or some of the samples (matching clusters may not be found in all samples). We refer to each row in this cluster match table as a sub-repertoire.
3. Differential abundance testing: sub-repertoires that exist in at least x number of samples per group are first selected (default is in at least 3). A sub-repertoire abundance matrix is then generated for the selected sub-repertoires, which contains the abundance of each sub-repertoire in each sample. This sub-repertoire abundance matrix can be generated in different ways. Typically, the original samples are first normalized to same total CDR3 sizes (abundances). Then, the abundance of a sub-repertoire in a sample is calculated as the sum of CDR3 counts belonging to that sub-repertoire in the sample; or the relative frequency of that sum is used as a relative abundance estimate; or to avoid bias in the previous two abundance estimates that might arise due to differences in the number of CDR3s per sub-repertoire in a sample, the relative clone size in sub-repertoires is used as a proxy for abundance (i.e., average clone size in sub-repertoire / average clone size in sample). Next, for each sub-repertoire, differential abundance testing between the two groups of samples is performed using various tests. We used t-test, and two-class, unpaired, RankProd [44] test, both of which work well. The CDR3 sequences belonging to significantly differentially abundant (DA) sub-repertoires are then extracted from each sample; these are candidate DA CDR3 sequences.
4. Filtering candidate DA CDR3s: DA sub-repertoires are not “pure” and contain CDR3s that are not necessarily associated with the condition thus requiring further filtering. To do this, all candidate DA CDR3 sequences (i.e., all CDR3 sequences that are in DA sub-repertoires) are first ranked as follows. For each candidate CDR3 *i*, a rank sum, C_*i*_, is computed by adding the candidate’s ranks from 6 factors:
  $$C_{i} = \mathop \sum \limits_{k = 1}^{6} R_{ki}$$
  where R_1*i*_ is candidate *i*’s importance in classifying the groups (random forest mean decrease in accuracy [45, 46]), R_2*i*_ is its mean fisher’s exact test p value calculated by comparing its abundance in each paired samples separately if data is paired/matched, or mean of mean fisher’s exact test p values calculated by comparing its abundance in a sample to every sample in the other group if data is unpaired/unmatched, R_3*i*_ is its mean odds ratio from the fisher’s exact test calculated for R_2_ with paired and unpaired samples handled as in R_2_, R_4*i*_ is its mean estimated increase in nucleotide to amino acid (nt-to-aa) ratio in the condition group compared to the control group (nt-to-aa in condition / nt-to-aa in control) to account for its level of convergent selection, calculated as a mean of such values obtained for it from each paired samples separately if data is paired/matched, or as mean of mean such values obtained for it by comparing its nt-to-aa in each sample to every sample in the other group if data is unpaired/unmatched, R_5*i*_ is the difference in the number of samples per group in which it exists, to account for its degree of condition induced “public-ness”, and R_6*i*_ is the number of times it has been detected in repeat resample runs of the DA analysis (Repseq datasets are huge datasets and require a lot of computational resources, we thus perform repeat sub-sampling of the raw datasets and run the differential abundance analysis of steps 1–3 for each resampled datasets, the candidate DA CDR3 sequences from each round are collected, CDR3 sequences that have been detected as candidate in multiple repeat resamples are given higher rank, i.e., rank of 1). The ranking in each factor is defined differently for assessing enrichment and de-enrichment. When assessing enrichment highest rank is given for highest mean decrease in accuracy for R_1,_ smallest p value for R_2_, highest odds ratio for R_3_, highest nt-to-aa ratio for R_4_, highest increase in detection in the condition/treatment group for R_5_, and highest number of detection in multiple runs of the analysis for R_6_. For assessment of de-enrichment, highest rank is given for highest mean decrease in accuracy for R_1,_ smallest p value for R_2_, lowest odds ratio for R_3_, lowest nt-to-aa ratio for R_4_, highest decrease in detection in the condition/treatment group for R_5_, and highest number of detection in multiple runs of the analysis for R_6._ The minimum value of 1 in each factor R signifies high rank. Since the range of rank values is different for each rank type, all Rs are scaled to be between 0 and 1 by subtracting the minimum and dividing by the range. We then calculate the p value for C_*i*_ using a randomization test, as the proportion of n rank sum values, calculated from n permutations (random shuffling of all Rs from the 6 factors, we typically used n = 1000), that are equal or less than C_*i*_. We consider candidate DA CDR3s with C p values less than 0.05 and q-value (minimal FDR at each p value) less than 0.05 as differentially abundant CDR3s. All six ranking factors were given equal weight in our analysis in the calculation of the rank sum C, but different weights could be used for each factor depending on the application. For false discovery rate estimation, we used a decoy-based strategy by including in the analysis randomly drawn CDR3 sequences from a reference database of healthy TCR CDR3 PBMC repertoires to each sample’s repertoire data. The rate of false detection of the decoy CDR3s was used to estimate the FDR and q-value at each p value level for all candidate CDR3s ordered from smallest to highest C p values.

### Datasets for testing the method

We used four TCR CDR3β immune repertoire datasets to test the method (Additional file 1: Table 1S). Celiac disease (CD) PBMC (n = 4) and Gut (n = 5) datasets of our celiac disease study cohort [9], yellow fever vaccination (YFV) PBMC dataset from DeWitt et al*.* (n = 9) [13] obtained from the public immune repertoire database, immuneACCESS, of Adaptive Biotechnologies (immuneACCESS, Adaptive Biotechnologies, Seattle, WA. Available from: http://adaptivebiotech.com/pub/dewitt-2015-jvi), and twin yellow fever vaccination (twinYFV) PBMC dataset (n = 6) from Pogorelyy et al*.* [16] obtained from their github (https://github.com/mptouzel/pogorelyy_et_al_2018).

### Method application on test datasets

We applied the method in the following manner for the celiac disease TCR CDR3β repertoire datasets in this work: We filtered out clonotypes with a size of 1, then, (1) 100 runs of steps 1 to 3 of the pipeline using randomly selected subsamples of 5000 unique CDR3β sequences for each sample. (2) Within sample CDR3β clustering using either nucleotide 4-mers or amino acid 3-mer feature vectors, and across sample cluster matching using k-means in step 2. (3) Sub-repertoire abundance matrix was generated by summing the abundance of CDR3s belonging to each sub-repertoire in each sample. Sub-repertoire level differential abundance detection was performed on sub-repertoires that exist in at least 3 samples per group using paired t-test with the p value cut-off of 0.1. We found from evaluations of results from multiple runs that the less stringent p value cut-off for comparing sub-repertoire abundances across samples helps increase the signal to noise ratio by providing the right level of “zooming-in”, without compromising detection capacity in downstream steps of the pipeline. 4) Combining the candidate CDR3βs from the 100 runs and performing the filtering step, CDR3βs with p value and q-value less than 0.05 were then considered condition-associated CDR3β sequences. The method was applied similarly for the huge yellow fever vaccination datasets (YFV and twinYFV) with the following changes: a) performed 600 runs of steps 1 to 3 (instead of 100, to allow exhaustive assessment of all clonotypes in all samples) with subsamples of 5000 CDR3β sequences per sample in each run, b) and performed only nucleotide 4-mer based analyses. The selected numbers for repeated runs of steps 1 to 3 of the method with down-sampled data allow exhaustive assessment of nearly all clonotypes in each dataset (Additional file 1: Fig. 8S).

Detailed description of the datasets, benchmarking, and characterization of detected condition associated CDR3s is given in the supplementary information section in Additional file 1.

## Supplementary Information

**Additional file 1**. Supplementary Information, Tables and Figures.

**Additional file 2**. Complete list of all the detected condition-associated CDR3s from all four datasets.

## Data Availability

The CD PBMC and CD Gut datasets are available as example datasets in the R implementation of the method which is at https://github.com/Greco-Lab/RepAn. The YFV PBMC dataset can be accessed from immuneACCESS (available at: http://adaptivebiotech.com/pub/dewitt-2015-jvi). The twin YFV PBMC dataset can be obtained from Pogorelyy et al*.* publication’s github repository (https://github.com/mptouzel/pogorelyy_et_al_2018).

## Abbreviations

CD: Celiac disease
CD Gut: The celiac disease gut biopsy repertoire dataset used in the study
CD PBMC: The celiac disease PBMC repertoire dataset used in the study
CDR1: Complementarity-determining regions 1
CDR2: Complementarity-determining regions 2
CDR3: Complementarity-determining regions 3
CDR3β: Complementarity-determining regions 3 of the beta chain
FDR: False discovery rate
HLA: Human leukocyte antigen
MHC: Major histocompatibility complex
pMHC: Peptide bound to Major histocompatibility complex
RepAn: Repertoire differential abundance analysis, the name of the method being reported
RepSeq: Deep, next generation repertoire sequencing
TCR: T-cell receptor
TRBD: T-cell receptor beta chain diversity gene
TRBJ: T-cell receptor beta chain joining gene
TRBV: T-cell receptor beta chain variable gene
twin YFV PBMC: The twin yellow fever vaccination repertoire dataset used in the study
YFV PBMC: The yellow fever vaccination repertoire dataset used in the study
YFV: Yellow fever virus
β-chain: Beta chain
