# Supplementary material for: Clustering based approach for population level identification of condition-associated T-cell receptor β-chain CDR3 sequences
Source: BMC Bioinformatics. 2021 Mar 25;22:159. doi: 10.1186/s12859-021-04087-7 (PMC7993519; doi:10.1186/s12859-021-04087-7)
Supplement: Supplementary file 1 — Additional file 1. Supplementary Information, Tables and Figures. [file 12859_2021_4087_MOESM1_ESM.pdf]

Supplementary Materials

Clustering based approach for population level identification of condition-associated T-cell receptor β-chain CDR3 sequences

Dawit A. Yohannes[1,2], Katri Kaukinen[3], Kalle Kurppa[4], Päivi Saavalainen[1,2,†], Dario Greco[5,6,7,†,*]

[1] Research Programs Unit, Translational Immunology, University of Helsinki, Helsinki, Finland

[2] Department of Medical and Clinical Genetics, University of Helsinki, Helsinki, Finland

[3] Department of Internal Medicine, Tampere University Hospital and Faculty of Medicine and Health Technology, Tampere University, Tampere, Finland

[4] Department of Pediatrics, Tampere University Hospital and Center for

Child Health Research, Tampere University, Tampere, Finland.

[5] Faculty of Medicine and Health Technology, Tampere University, Tampere, Finland

[6] BioMediTech Institute, Tampere University, Tampere, Finland

[7] Institute of Biotechnology, University of Helsinki, Helsinki, Finland

† These authors contributed equally to this work

* Corresponding Author: dario.greco@tuni.fi

Supplementary Information

Datasets for testing the method

The sample preparation and sequencing of the datasets have been described before (1,2); in brief, genomic DNA was extracted from total PBMC for Celiac disease patients in CD PBMC dataset, and yellow fever virus vaccination volunteers in YFV PBMC dataset, or from gut biopsy for celiac disease patients in CD Gut dataset. The TCR CDR3β region was then deeply sequenced using the ImmunoSeq assay (3) (www.adaptivebiotech.com ; www.immunoseq.com), which uses optimized multiplex PCR to amplify the TCR CDR3β region, and Illumina for sequencing. It then determines the CDR3β sequences and their abundance, as well as annotates the gene segments according to International ImMunoGeneTics (IMGT) specifications (4). Sample preparation information for the twin YFV dataset is available in Pogorelyy *et al.* (5), briefly 3 twin pairs have been immunized for YFV and their TCR repertoire sequenced and processed using MiXCR (6). We used the day 0 and day 15 repertoires from the twin YFV data, importantly, these TCRs come from cDNA and not from gDNA and only show expressed TCR counts in the samples.

Characterization of differentially abundant CDR3β sequences

To evaluate TRBV gene usage and per position amino acid usage among differentially enriched CDR3β sequences, we compared the observed usage frequencies in the list of enriched CDR3βs to the frequencies obtained from 100 randomly sampled sets of CDR3βs (same size as the enriched list) from the combined dataset off all samples. The significance of the observed frequencies was calculated as the proportion of frequencies in the randomly sampled sets that were equal or more than the observed frequencies (p-values less than 0.05 were considered statistically significant). The per position amino acid usage analysis was performed for selected TRBV genes (that showed statistically significant over-usage in the differentially enriched TCRs) by comparing observed frequencies to frequencies obtained from randomly sampled sets using the same TRBV gene and CDR3 length.

Analysis of same condition samples

To assess the method’s robustness in detecting condition relevant clonotype abundance differences as opposed to mere sampling variation, we compared randomly drawn same condition samples of

CD PBMC for both day 0 and day 6 conditions (Figure 5S). In each case, samples belonging to each condition were first pooled. From the pooled repertoire, 8 samples (of size 1/4th of the total pooled repertoire) were randomly drawn in a probabilistic manner with high abundance clonotypes having higher probability of being sampled (sample function in R, with replacement). In each randomly drawn sample, the number of times a clonotype was observed was taken to be its abundance. To simulate differences in clonotype size and antigen exposure history, the abundance estimate for 10% of the randomly drawn clonotypes was increased by a randomly selected factor of 20 to 400% (with equal probability). The nt 4-mer based analysis was then performed on the 8 randomly drawn samples with 4 samples each assumed to come from different conditions (even though they were drawn from same condition pool). This analyses was done ten times for both day 0 and day 6 conditions, and the number of clonotypes detected as enriched reported (Figure 5S).

Enrichment analysis of known condition-associated CDR3βs

To evaluate the effectiveness of the method in identifying CDR3βs that are truly associated with a condition, we compared the number of observed CDR3βs known to be associated with the condition (from previous studies), to the frequencies of such known CDR3βs obtained from 100 random sets of CDR3βs (same size as the enriched list) sampled from the combined dataset of all samples. The significance of the observed frequency of known CDR3βs was calculated as the proportion of known CDR3βs in the randomly sampled sets that were equal or more than the observed frequency. Alternatively, the proportion of known CDR3βs in the list of enriched CDR3βs was compared to the proportion of known CDR3βs in the total combined repertoire of samples in a dataset using fisher's exact test with p-values less than 0.05 considered statistically significant.

Benchmarking

We compared the performance of the method to four recently published methods for celiac-associated CDR3 detection in the CD PBMC dataset. The methods are vdjRec (7), DeWitt's method (2), and Alice (8),and our previously published method here referred to as the Yohannes method (1). The methods are different in their design and application, Yohannes's method (1) and vdjRec (7) allow comparison at the population level but work only on public CDR3s (and thus identify only public condition associated CDR3s). Conversely, DeWitt's method (2) and Alice (9) allow comparison only at individual level but allow detection of both private and public CDR3s. Our

current method, here referred to as RepAn, allows direct population level comparison between groups of samples for detection of both private and public CDR3s. We also compared the method to results that are obtained when germline gene usage instead of k-mer frequency distance is used to cluster clonotypes in step 1 and step 2. As step 1 and step 2 are independent of subsequent steps, we aimed to evaluate the performance of kmer based clustering as opposed to simple germline gene usage grouping of clonotypes in the final outcome.

Input data for vdjRec and Alice for every V-J combination in CD PBMC datasets was prepared, and estimation of CDR3 generation probability was performed as described by the methods. Both vdjRec and Alice were run only on the four gluten exposed day 6 samples of CD PBMC. DeWitt's method was implemented in R and was run on each sample pair with minimum total count cutoff of 100 per CDR3. For the individual level only methods, we collected the union of all identified CDR3s across all individuals as CD associated, this entails that some CDR3s that have contradictory enrichment tendencies in different individuals would be considered as condition associated while population level approaches may not consider such CDR3s as condition-associated. On the other hand, population level methods require a strong signal in multiple subjects to detect condition associated CDR3s, thus may miss CDR3s with low level signal or that are highly private.

Since there is no exhaustive truth set of CD associated CDR3s, we compared the methods in two ways. We first looked at how many of the 56 previously published, well-known CD-associated CDR3s present in the total CD PBMC dataset the methods detect. The 56 celiac disease associated CDR3s in our dataset are among those published by Qiao *et al.*, Han *et al.* and Petersen *et al.* as gluten tetramer reactive CDR3βs, thus are a suitable truth set for comparing the methods (10–12). Second, we assessed the methods' Recall (True positives / (True positives + False negatives)) and Precision (True positives / (True positives + False Positives)) metrics. As we only really have three methods that can detect both private and public clonotypes, namely, our method RepAn, ALICE and Dewitt's method, and the concordance of detected enriched clonotypes by any two methods rather low, we defined true positives (TP) as clonotypes detected as enriched by the method under consideration and by at least one other method, false positives (FP) as clonotypes detected by the method under consideration and by no other method, and false negative (FN) as clonotypes not detected as enriched by the method under consideration but detected as enriched by at least two other methods. We assessed recall and precision separately for all variants of RepAn against the other tools.

Supplementary Figures

Table 1S: The three datasets used in the study. The number of total and unique nucleotide CDR3β sequences is shown for each sample.

Dataset	Sample name	HLA	Group 1		Group 2	
Celiac Disease (CD) (PBMC, n=4)			Day 0 (before eating wheat gluten)		Day 6 (After eating wheat gluten for 3 days)	
			Productive Total CDR3	Productive unique	Productive Total	Productive unique
	CD005	DQ2/DQ2	527382	16174	589603	24195
	CD006	DQ2/DQ5	794514	13013	582690	15179
	CD011	DQ2/DQ8	541814	16014	384155	10452
	CD039	DQ2/DQ2.2	339279	10221	436176	10403
Celiac Disease (CD) (Gut, n=5)			GFD (after 1 year gluten free diet treatment)		ACT-disease (Sampled right after diagnosis)	
			Productive Total	Productive unique	Productive Total	Productive unique
	CD1GB	DQ2/DQ6	1189542	5886	1233903	10434
	CD2GB	DQ2/DQ6	1760963	10033	968548	11715
	CD3GB	DQ2/DQ6	3339752	8284	1101307	9863
	CD4GB	DQ2/DQ2	2197571	9567	757564	10423
	CD5GB	DQ2/DQ2	761406	6266	830042	5370
Yellow Fever Vaccine (YFV) immunization data (PBMC, n=9)			Day 0 (pre-vaccination of single dose of YF-17D)		Day 14 (post-vaccination)	
			Productive Total	Productive unique	Productive Total	Productive unique
	Subject 1		5520003	334966	9034146	365705
	Subject 2		7546126	385506	9535753	434828
	Subject 3		4351030	339330	8930117	348878
	Subject 4		4522888	244022	8343948	292385
	Subject 5		1281823	64751	9201942	316705
	Subject 6		4525600	350914	6873009	275193
	Subject 7		3672998	173178	25873400	222176

	Subject 8		4761179	277491	9991028	258036
	Subject 9		3533232	207966	9110762	228819
Twin Yellow Vaccine (YFV) immunization data (PBMC, n=6)			Day 0 (pre-vaccination YF-17D)		Day 15 (post-vaccination)	
			Productive Total	Productive Unique	Productive Total	Productive Unique
	P1F1		866708	620215	1822686	971438
	P2F1	A-02:01:01 / A-02:01:01	995142	651322	2234188	1266196
	Q1F1	A-02:01:01 / A-02:01:01	824792	386868	867196	473610
	Q2F1	A-02:01:01 / A-02:01:01	1426320	729546	1488912	645733
	S1F1	A-02:01:01 / A-02:01:01	952932	570846	1112417	722045
	S2F1	A-02:01:01 / A-03:01:01	1388145	756816	1662225	887756

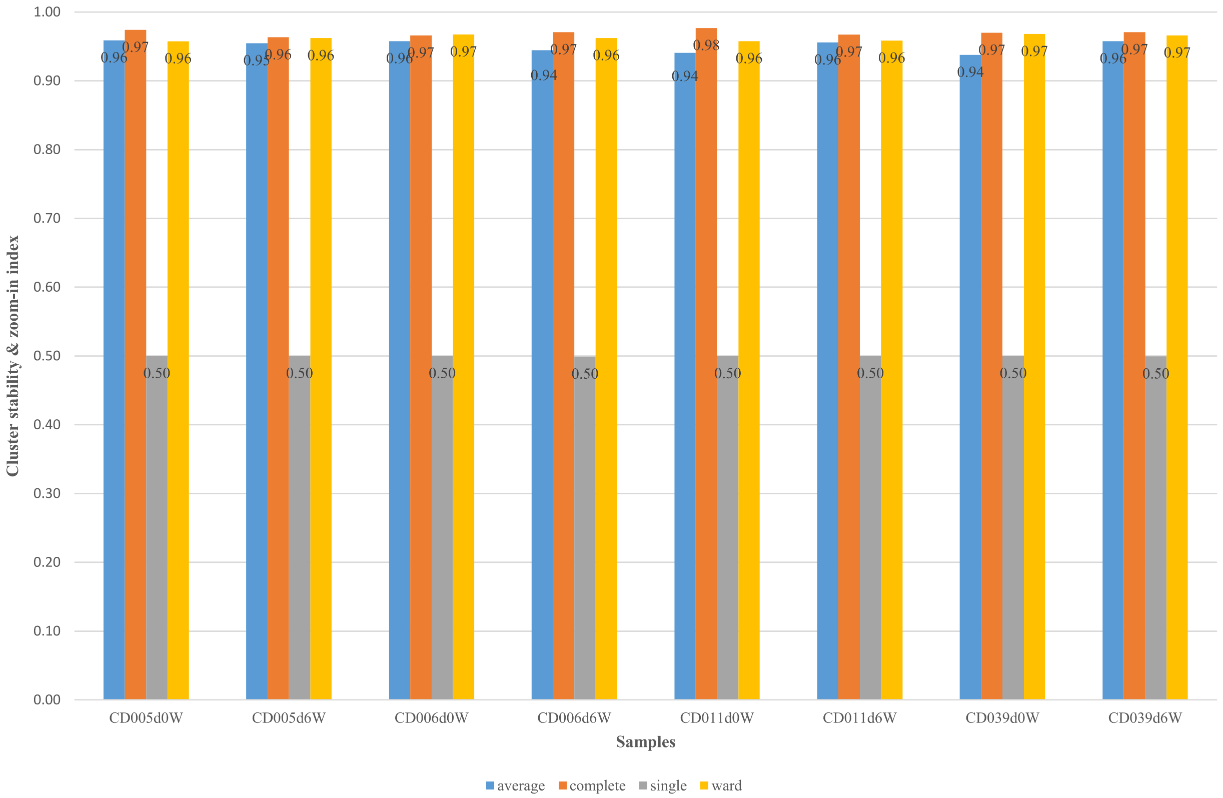

Figure 1S: Comparison of linkage methods in the hierarchical clustering of TCR CDR3s. Linkage methods were evaluated using a combined measure of cluster stability (the cls.stab.sim.ind in R package clv (13), *using the Rand similarity index), and a zoom-in factor (1-(average cluster size)/(total number of CDR3s)), favoring smaller sized clusters relative to the total number of starting CDR3s that allow deeper zooming into the diverse repertoire samples. We sum the two values and divide by 2 to get a stability & zoom-in index between 0 and 1, with 1 meaning stable clustering with high number of small sized clusters. We resampled 5000 unique TCR CDR3 sequences from all CD PBMC samples and evaluated the clustering performance of commonly used linkage methods in hierarchical clustering: average, complete, ward, and included the single linkage method. In each case, we performed hierarchical clustering of the CDR3s, partitioned the CDR3s into k clusters as determined by the dynamic tree cut algorithm* (14), *and evaluated stability at the determined k number of clusters. The complete linkage method performed consistently better for all samples followed by the ward method. We chose to use the complete method because it gave good stability with more number of clusters, allowing for deeper "zooming-in", which is critical as it reduces repertoire diversity more.*

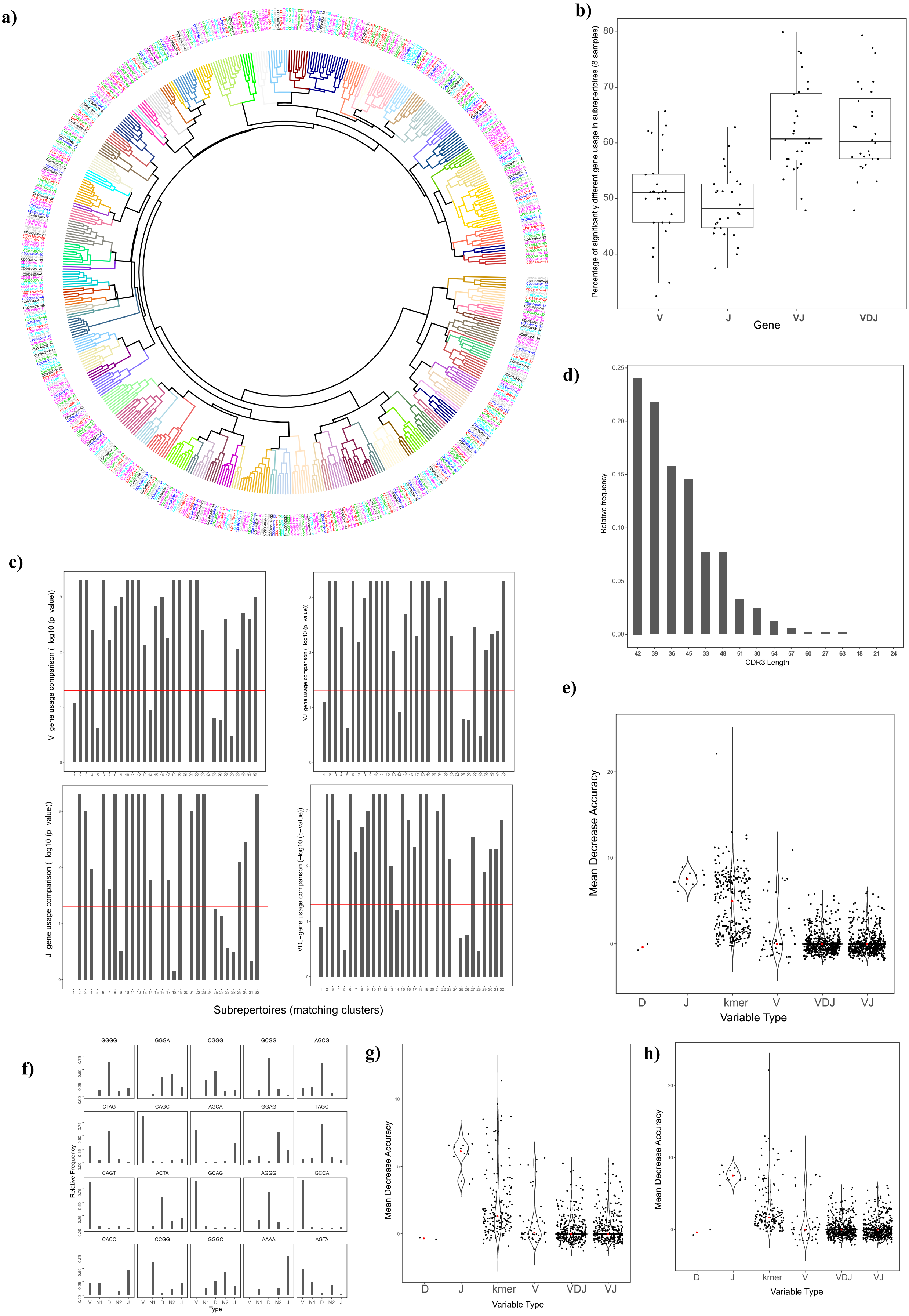
a)
b)
Percentage of significantly different gene usage in subrepertoires (8 samples)
Gene
V
J
VJ
VDJ
c)
V-gene usage comparison (−log10 (p−value))
VJ−gene usage comparison (−log10 (p−value))
J-gene usage comparison (−log10 (p−value))
VDJ−gene usage comparison (−log10 (p−value))
Subrepertoires (matching clusters)
d)
Relative frequency
CDR3 Length
e)
Mean Decrease Accuracy
Variable Type
D
J
kmer
V
VDJ
VJ
f)
GGGG
GGGA
CGGG
GCGG
AGCG
CTAG
CAGC
AGCA
GGAG
TAGC
CAGT
ACTA
GCAG
AGGG
GCCA
CACC
CCGG
GGGC
AAAA
AGTA
Relative Frequency
Type
V
N1
D
N2
J
g)
Mean Decrease Accuracy
Variable Type
h)
Mean Decrease Accuracy
Variable Type

Figure 2S: CDR3 sub-repertoire matching in samples of unrelated individuals. (a) hierarchical clustering of CDR3 cluster centroids from all CD PBMC samples is shown. Samples are indicated in different colors. Branch colors indicate sub-repertoires. All detected 82 sub-repertoires contained clusters from more than one sample. (b) V-, J-, VJ- and VDJ gene usage frequency was compared in all sub-repertoires between clusters coming from pairs of samples, and percentage of sub-repertoires with significantly different usage indicated (chi-square test of independence p-value below 0.05). This was done for all the possible 28 unique pairs of samples and shown in the boxplots. (c) For each of the 32 sub-repertoires of samples CD005 and CD006, V-,J-, VJ- and VDJ gene usage frequency was compared between clusters coming from the two samples, the dotted line indicates the cut-off point at p-value 0.05 (using chi-square test of independence) in –log10(p-value) above which the gene usage is significantly different. (d) The relative frequency of all CDR3 lengths (in nucleotide) in a single CD PBMC repertoire is shown. The result was the same for all other samples. The most used CDR3 length was 42 nucleotides or 14 amino acid long. (e) The classification importance of k-mers and genes in distinguishing 4-mer based sub-repertoires across the 8 samples is shown. (f) The frequency of where (in V, N1, D,N2, J) the top 20 most discriminative 4-mers (ordered left to right, top to bottom) are found in all CDR3s of all 8 samples is shown.(g) for sample CD005 and (h) for all the 8 CD PBMC samples show the classification importance score distributions similar to the plot on (e) using only k-mers primarily originating (most frequently) from any of the N1,D and N2 regions (which were 157 and 154 k-mers out of the 256 4-mers respectively).

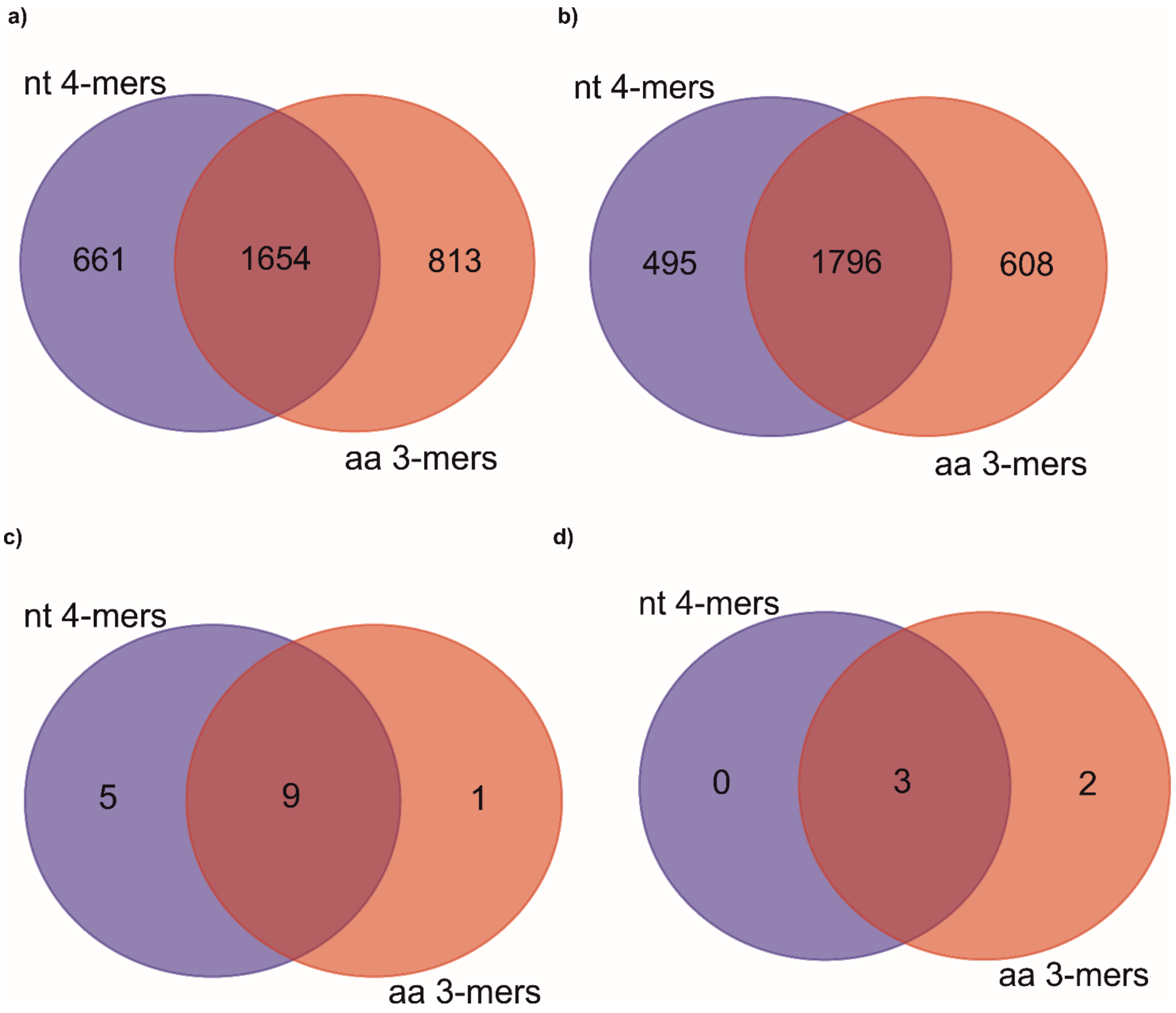

Figure 3S: The overlap between the differentially enriched CDR3β sequences of the DA analyses using nt 4-mer and aa 3-mer feature vectors is shown for (a) CD PBMC and (b) CD Gut datasets.The overlap between the known CD-specific clonotypes detected by the nt 4-mer and aa 3-mer approaches is shown for (c) CD PBMC and (d) CD Gut.

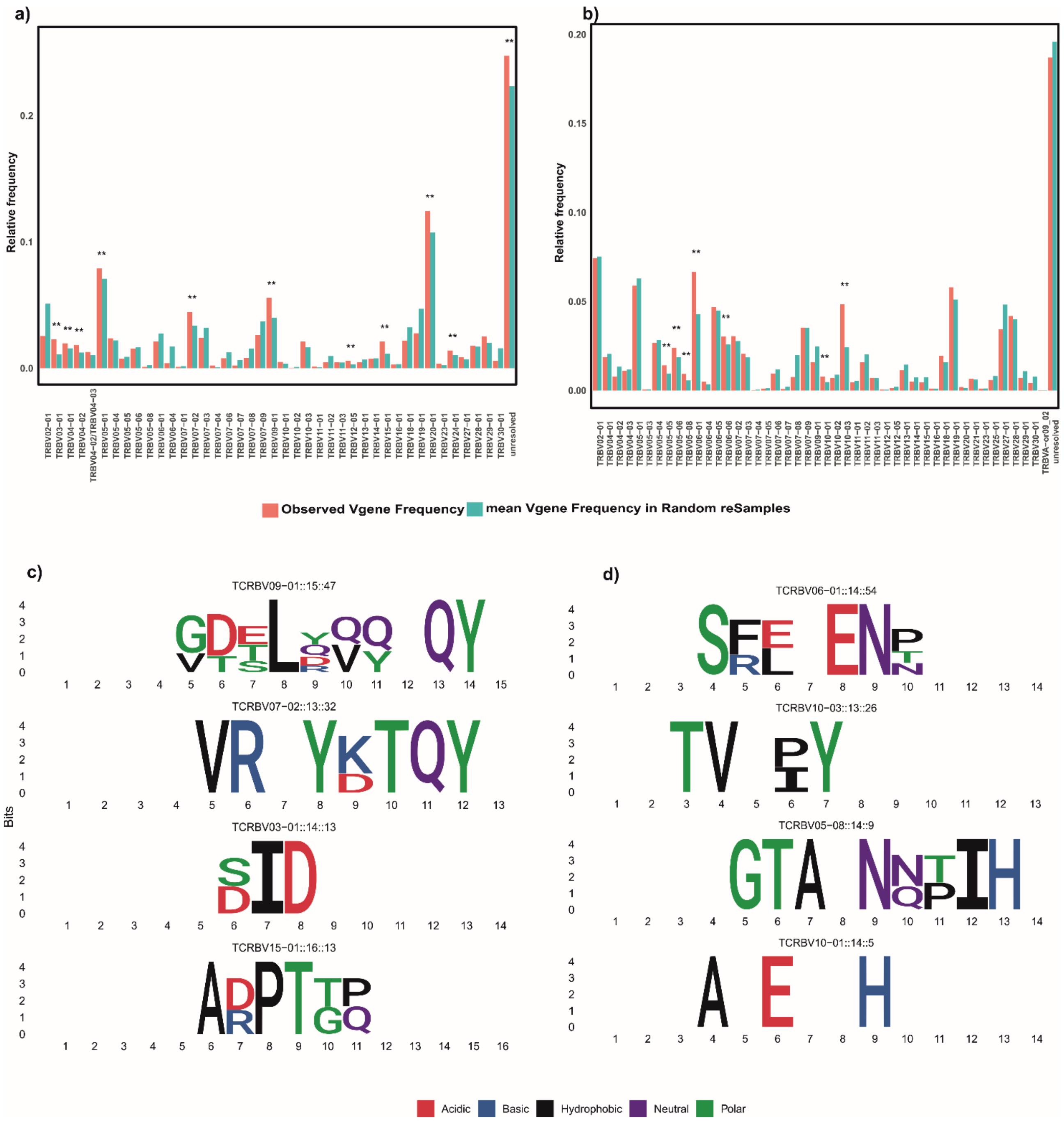

Figure 4S: Characteristics of the differentially abundant CDR3b sequences in CD PBMC and CD Gut. The differentially enriched CDR3b sequences had biased usage of TRBV genes that are known to be over-represented in gluten reactive CDR3b sequences in previous studies, such as TRBV07-02 and TRBV09-01 from CD PBMC (a), and TRBV07-09 from CD Gut (b) (observed frequencies are shown in red, mean frequency from randomly generated sets of CDR3s are shown in blue). Significantly, over-used amino acids at each position are shown for the enriched CDR3β sequences that use TRBV genes detected to be over-used from CD PBMC (c) and CD Gut (d), amino acids are colored according to their properties. The information content of significantly overused amino acids at each position is shown in bits on the y-axis. TRBV and per-position amino acid over-usage

is assessed by comparing the observed frequencies in the set of differentially enriched CDR3s to that obtained by chance in 100 randomly sampled CDR3s of same size, TRBV gene and CDR3 length, with p<0.05 considered significant (gene names indicate TRBVgene::CDR3 length::number of CDR3s in the enriched list with the Vgene and CDR3 length). The results from using aa 3-mer feature vectors are shown.

a)

Condition	# detected as Enriched	Known CD associated CDR3s
day 0	0	0
day 0	3	0
day 0	0	0
day 0	4	0
day 0	2	0
day 0	1	0
day 0	2	0
day 0	1	0
day 0	1	0
day 0	1	0
day 6	2	0
day 6	1	0
day 6	1	0
day 6	1	0
day 6	3	0
day 6	0	0
day 6	7	0
day 6	5	0
day 6	3	0
day 6	1	0

b)

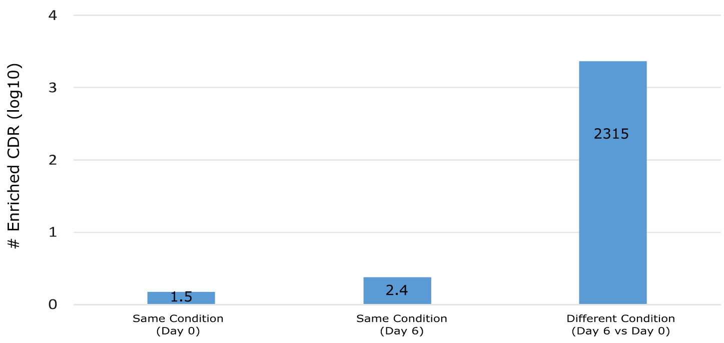

Figure 5S: Application of method on same condition samples. The method (nt 4-mer approach) was applied on eight randomly drawn samples from the pooled repertoire of CD PBMC for day 0 and day 6 separately (with n=4 per group). The robustness of the method to clonotype abundance differences due to sampling variation was evaluated. (a) The analyses results from ten "pooling - random sampling" experiments for day 0 and day 6 condition is shown. (b) The average number of CDR3s detected as enriched for day 0 and day 6 same condition analyses is shown side by side with the result obtained for the CD PBMC dataset when day 6 is compared to day 0 samples (in log10 for better visualization, actual numbers are shown on the bars).

Table 2S: List of previously known gluten-reactive celiac disease associated CDR3b sequences identified as differentially enriched by the method from the CD PBMC repertoire dataset.

	pre-gluten challenge (day 0)				post-gluten challenge (day 6)				Clustering based DA analysis result output													
	CD005d0W	CD006d0W	CD011d0W	CD039d0W	CD005d6W	CD006d6W	CD011d6W	CD039d6W	subRep_resampleRun	resampleRank	rfRank	ntaaRank	fpval	fPvalRank	fOr	fOrRank	nSamRank	rpRanks	permutedEnPval	RorDecoy	FDRfromDecoy	qvalue
**Using nt 4-mers/Enriched**																						
CASSLRSTDTQYF	13	0	0	0	613	15	0	462	53_18;93_2	10	24	5	1.33E-08	2877	188.699989	360	3	1	0.000999	Real	0	0
CASSLNWDTEAFF	0	0	0	0	0	154	0	74	12_13;72_1	13	23	23	2.63E-18	1906	168.0506677	418	3	2	0.000999	Real	0	0
CASSFRSTDTQYF	0	0	0	0	0	415	0	441	53_18;93_2	15	23	76	6.61E-108	263	226.5555174	289	3	7	0.000999	Real	0	0
CASSIRHTDTQYF	0	0	0	0	0	102	0	220	53_18;93_2	17	23	23	1.52E-37	1161	191.2283889	353	3	4	0.000999	Real	0	0
CASSLRHTDTQYF	0	0	0	0	285	0	0	0	53_18;85_2	21	23	9	6.24E-113	240	369.8323347	130	4	5	0.000999	Real	0	0
CASSVRFTDTQYF	0	0	0	0	0	0	49	0	93_24;18_4	22	23	9	9.53E-30	1408	112.8709438	591	4	12	0.000999	Real	0	0
CASSLRSGDTQYF	0	0	0	0	140	0	0	42	22_15;93_2	23	23	76	1.88E-10	2579	80.3744431	716	3	588	0.010989	Real	0.00211	0.00065
CASSLRFTDTQYF	0	0	0	0	14	0	0	0	93_24	28	23	9	4.31E-07	3200	25.17366849	1130	4	632	0.011988	Real	0.001783	0.00065
CASSIRWTDTQYF	0	0	0	0	0	124	0	0	53_18;18_4	25	23	76	2.44E-45	952	169.2548451	412	4	586	0.016983	Real	0.001359	0.00065
CASSLGGQLFF	0	0	0	0	0	0	0	166	15_41;30_4	25	23	76	8.37E-41	1062	131.640938	525	4	677	0.016983	Real	0.001355	0.00065
CASSIRATDTQYF	0	0	0	0	0	23	0	72	18_45;39_4	25	23	76	8.84E-09	2841	44.91550287	890	3	1202	0.021978	Real	0.001014	0.00065
CASSLGETQYF	20	102	39	0	103	76	19	24	50_12;30_1	7	23	76	0.048454	5814	4.200017855	2585	4	853	0.027972	Real	0.000798	0.00065
CASSPGVYEQYF	0	0	0	0	0	0	59	0	40_35;50_4	25	23	76	6.50E-22	1721	83.59026353	704	4	1144	0.033966	Real	0.001291	0.001135
CASSLASAGGTDTQYF	0	0	0	0	0	39	0	0	60_6;85_25	25	23	76	3.23E-14	2200	54.11666221	841	4	1576	0.042957	Real	0.004613	0.001811
**Using aa 3-mers/Enriched**																						
CASSLRSTDTQYF	13	0	0	0	613	15	0	462	77_10;15_1	3	23	4	1.33E-08	2906	188.4787478	370	3	1	0.000999	Real	0	0
CASSIRHTDTQYF	0	0	0	0	0	102	0	220	58_4;15_12	13	23	31	1.49E-37	1174	191.1018268	362	3	2	0.000999	Real	0	0
CASSLNWDTEAFF	0	0	0	0	0	154	0	74	62_23;35_2	23	23	31	2.71E-18	1920	168.0688056	426	3	12	0.000999	Real	0	0
CASSLRHTDTQYF	0	0	0	0	285	0	0	0	37_14;7_21	25	23	10	6.41E-113	251	369.4179069	131	4	4	0.000999	Real	0	0
CASSVRFTDTQYF	0	0	0	0	0	0	49	0	58_4;8_33;	25	23	10	1.17E-29	1423	112.3818586	601	4	13	0.000999	Real	0	0
CASSIRWTDTQYF	0	0	0	0	0	124	0	0	58_4;15_12	22	23	86	2.37E-45	966	169.3233377	421	4	99	0.001998	Real	0	0
CASSLRFTDTQYF	0	0	0	0	14	0	0	0	37_14	31	23	10	4.31E-07	3240	25.14544733	1138	4	537	0.012987	Real	0	0
CASSIRATDTQYF	0	0	0	0	0	23	0	72	58_4;37_14	26	23	86	8.79E-09	2875	44.88547726	901	3	773	0.018981	Real	0	0
CASSLGGQLFF	0	0	0	0	0	0	0	166	30_69;7_78	29	23	86	8.87E-41	1076	131.4722929	534	4	891	0.024975	Real	0	0
CASSLTASNQPQHF	0	0	0	0	89	0	0	0	3_49;70_61	30	23	86	4.36E-24	1641	80.8306283	725	4	1635	0.048951	Real	0.000415	0.000175

- Numbers for each sample indicate CDR3 abundance in counts per million.
- CDR3s are always shown in amino acid sequence even when the DA analysis is performed using nucleotide level k-mers as feature vector
- subRep_resampleRun: the subrepertoire label to which the CDR3 belongs in the particular resample Run.
- resampleRank: ranking of how often a CDR3 sequence is detected as DA in the repeat resample runs
- rfRank : ranking of random forest feature importance measure (mean decrease in classification accuracy)

- ntaaRank: ranking of the nucleotide to amino acid ratio (i.e the number of nucleotides encoding for the same amino acid). The ranking is done for the average ntaa ratio of the amino acid CDR3 sequence across the samples in which it exists
- fpvalRank : ranking of the fisher exact test p-value obtained from the comparison of the frequency of the CDR3 sequence between the two samples from the same patient (e.g CD005 day 0 sample versus Cd005 day 6 sample). The ranking is done for the average p-value (column fpval) the CDR3 sequence attained from the individuals in which it exists.
- fOrRank : ranking of the fishers exact test odds ratio obtained from the comparison of the frequency of the CDR3 sequence between the two samples from the same patient (e.g CD005 day 0 sample versus Cd005 day 6 sample). The ranking is done for the average odds ratio (column fOr) the CDR3 sequence attained from the individuals in which it exists.
- nSamRank: ranking of the number of post-challenge (day 6) minus pre-challenge (day 0) samples in which the CDR3. (CDR3s appearing in more day 6 samples than day 0 samples are given higher rank)
- rpRank: the rank of the sum ranking values, called here rpRank or C in the main text, of the above 6 ranking factors
- permutedEnPval : the p-value calculated for CDR3s, as the proportion of rt (obtained by permutation of all rank factors 1000 times) that is less or equal to the observed rt for a CDR3. (Alternatively, is greater or equal to the observed rt for identifying significantly de-enriched CDR3s).
- qvalue: the q-value for the CDR3, which is the minimal FDR level (below p-value cut-off of 0.05) at which the CDR3 can be accepted as differentially abundant.
- See Supplementary dataset 1 for a full list of differentially enriched Celiac disease associated CDR3 sequences identified.

Table 3S: List of previously known gluten-reactive celiac disease associated CDR3b sequences identified as differentially enriched/de-Enriched by the method from the CD Gut repertoire dataset.

	After 1 year GFD treatment					During active celiac disease					Clustering based DA result output															
	CD1GBgfd	CD2GBgfd	CD3GBgfd	CD4GBgfd	CD5GBgfd	CD1GBact	CD2GBact	CD3GBact	CD4GBact	CD5GBact	subRep_resampleRun	resampleRank	rfRank	ntaaRank	fpval	fPvalRank	fOr	fOrRank	nSamRank	rpRanks	permutedEnPval	RorDecoy	FDRfromDecoy	qvalue
**Using nt 4-mers/Enriched**																										
CASSTGNQPQHF	0	0	0	0	0	0	126	0	0	0	62_46;32_95	46	6.00E+01	15	3.06E-66	2792	273.05	1207	4	235	6.99E-03	Real	0	0
CASSGGNQPQHF	0	274	0	0	0	0	521	0	0	0	40_1;58_5;8_19;42_	39	6.00E+01	15	9.36E-136	986	5.0016	3893	5	257	9.99E-03	Real	0	0
CASSLGYEQYF	0	54	0	0	0	53	61	44	87	0	10_9;19_23;51_47;	38	6.00E+01	39	3.71E-05	10688	147.47	1722	2	613	1.40E-02	Real	0	0
**Using nt 4-mers/DeEnriched**																										
CASSVRFTDTQYF	0	34	0	105	20	0	0	0	0	0	57_37;15_39;17_43	41	4.10E+01	1	2.43E-06	10047	0.0407	2571	1	1.74E+03	3.50E-02	Real	0	0
CASSLRSTDTQYF	0	64	0	208	95	0	0	0	19	0	74_25;57_37;15_39	41	4.10E+01	1	2.43E-06	10047	0.0407	2571	1	1.74E+03	0.034965	Real	0	0
**Using aa 3-mers/Enriched**																										
CASSGGNQPQHF	0	274	0	0	0	0	521	0	0	0	16_79;16_84;18_85	42	6.00E+01	13	9.18E-136	1001	5.0019	3930	5	238	1.20E-02	Real	0.00188	0.001255
CASSTGNQPQHF	0	0	0	0	0	0	126	0	0	0	31_87;54_94	50	6.00E+01	13	3.04E-66	2803	273.07	1210	4	225	1.20E-02	Real	0.001779	0.001255
CASSLGYEQYF	0	54	0	0	0	53	61	44	87	0	30_7;16_27;61_30;	39	3.70E+01	52	2.00E-01	13937	108.06	1944	2	634	1.40E-02	Real	0.003306	0.001255
CASSLGGELFF	0	0	0	0	0	670	0	0	0	0	13_9;52_40;32_48;	47	6.00E+01	140	4.45E-195	423	647.04	522	4	876	2.50E-02	Real	0.001708	0.001255
CASSIRSTDTQYF	0	130	141	208	84	0	0	303	74	52	54_5;36_8;63_11;6	5	4.40E+01	55	2.38E-01	14115	2.4352	5670	6	1488	3.40E-02	Real	0.001255	0.001255
**Using aa 3-mers/DeEnriched**																										
CASSLRSTDTQYF	0	64	0	208	95	0	0	0	19	0	55_3;38_15;55_28;	44	41	12	2.23E-11	8430	0.1012	2996	2	1756	4.80E-02	Real	0	0

Table 4S: Previously known condition-associated CDR3s in the list of DA enriched CDR3s identified by the method compared to the frequency of known CDR3s in the total combined dataset (using fisher's exact test)

**Dataset**	**Feature space**	**known condition-associated CDR3s in DA enriched CDR3s / total Enriched**	**known condition-associated CDR3s in all samples / Total clones in dataset**	**Fisher's exact test p-value**
CD PBMC	nt 4-mer	14 / 2315	56 / 115651	p= 8.367e-11
CD PBMC	aa 3-mer	10 / 2467	56 / 115651	p=1.129e-06
CD GUT	nt 4-mer	3 / 2291	45 / 87839	p= 0.1226
CD GUT	aa 3-mer	5 / 2404	45 / 87839	p=0.01049
YFV PBMC	nt 4-mer	697/ 2620	12092 / 2373394	p < 2.2e-16
Twin YFV PBMC	nt 4-mer	2058 / 4152	5730 / 1847053	p < 2.2e-16

Table 5S: Number of previously known published YFV-specific CDR3s detected in the list of DA enriched CDR3s identified by the method for Twin YFV PBMC dataset. All results here are based on exact matches (0 mismatch).

SampleName	CMV *	YFV *#	CMV	YFV#	CMV	YFV (FC > 32)+#
S2	0	5	0	19	0	11
S1	0	3	0	15	0	12
P2	0	1	0	14	0	9
P1	0	2	0	14	0	11
Q2	0	4	0	15	0	12
Q1	0	3	0	14	0	13

* Number of published clonotypes found in the enriched clonotypes reported by Pogorelyy, Minervina, Touzel, *et al.* (5).

+ For a fair comparison with the study by Pogorelyy, Minervina, Touzel, *et al.*, known clonotypes were checked in the enriched clonotypes produced by our method that had an abundance fold change of > 32 in day 15 compared to day 0 (this was the cutoff used in their study)

# Significantly more exact matches of published YFV-specific clonotypes were detected by our method compared to the reported by Pogorelyy, Minervina, Touzel, *et al.*, with t-test p-values of 5.978e-07 for our result with no FC cutoff and 4.212e-06 with FC cutoff.

Previously published YFV and CMF specific clonotypes were obtained from the original study (5).

Table 6S: Comparison to other published methods. The current method, RepAn, identifies 10 of the 56 CD-associated CDR3s (using nt 4-mers) that exist in the CD PBMC dataset as differentially enriched during gluten exposure.

**Method**	**Analysis type**	**Detected CDR3 type**	**# enriched CDR3s**	**# known CD CDR3s**	**proportion of knowns**	**TP**	**FP**	**FN**	**Recall TP/(TP+FN)**	**Precision TP/(TP + FP)**
RepAn (nt 4-mer)	Population level	public/private	2315	14	0.006	1105	1210	2218	0.90	0.48
RepAn (V-gene)	Population level	public/private	3495	12	0.003	1017	2478	2218	0.89	0.29
RepAn (VJ-gene)	Population level	public/private	2968	14	0.004	1024	1944	2218	0.89	0.35
RepAn (VDJ-gene)	Population level	public/private	2943	15	0.005	1022	1921	2218	0.89	0.35
RepAn (J-gene)	Population level	public/private	2745	12	0.004	863	1882	2218	0.87	0.31
ALICE	Subject level	public/private	151	9	0.060	41	110	4382	(0.08, 0.02, 0.02, 0.02, 0.03)	(0.27, 0.28, 0.30, 0.28, 0.31)
DeWittMethod	Subject level	public/private	2003	12	0.006	1118	885	2530	(0.91, 0.90, 0.90, 0.90, 0.88)	(0.56, 0.51, 0.52, 0.52, 0.43)
YohannesMethod	Population level	public	33	4	0.121	30	3	4500	(0.01, 0.02, 0.02, 0.01, 0.02)	(0.91, 0.97, 0.96, 0.91, 0.91)
vdjRec	Population level	public	31	1	0.032	15	16	4502	(0.007, 0.007, 0.007, 0.007, 0.009)	(0.48, 0.45, 0.52, 0.52, 0.52)

For ALICE, DewittMethod, YohannesMethod and vdjRec, TP, FP and FN values from only the comparison to RepAn with nt 4-kmers is given, Recall and Precision values are given for the separate comparisons with each variant of RePan in the order nt 4-mer, V-gene, VJ-gene, VDJ-gene, J-gene.

Table 7S: Memory and CPU run time requirements for the test runs of the method.

**Dataset**	**resample Rounds**	**CPU Time (hours)**	**Memory**
CD PBMC	100	1.14 (nt 4-mers), 14.63 (aa 3-mers)	96.6 GB *
CD Gut	100	0.95 (nt 4-mers), 8.62 (aa 3-mers)	93.3 GB *
CD PBMC	600	49.60 (nt 4-mers)	321.6 GB
CD PBMC	600	41.52 (nt 4-mers)	211.6 GB

* Memory estimates for the combined nt 4-mer and aa 3-mer analysis is given as both were run in the same batch job. It should be noted that the aa 3-mer analysis takes a significant portion of these memory used.

For all analysis, intermediate results from each resample round are written to file and accessed when needed to decrease memory requirements.

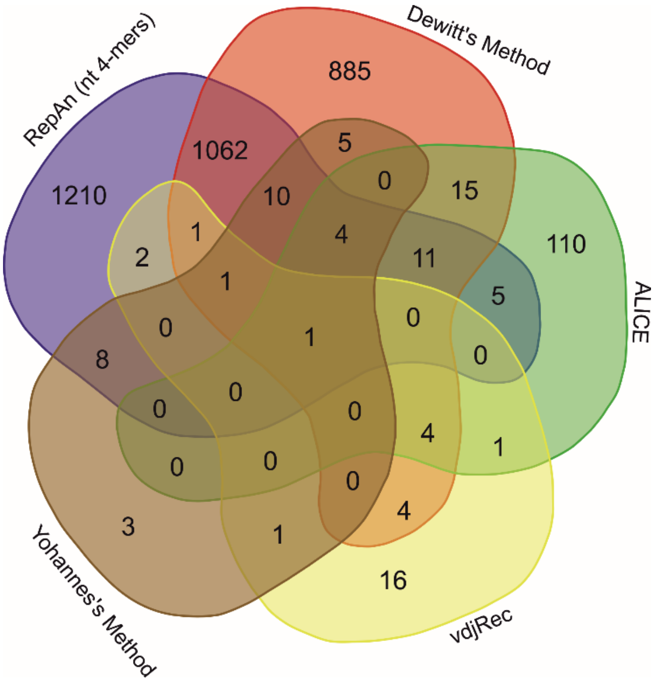

Figure 6S: The overlap between the differentially enriched CDR3β sequences of the CD PBMC dataset by our method, RepAn using nt 4-mer, Dewitt's method, ALICE, vdjRec, and Yohannes's method.

a)

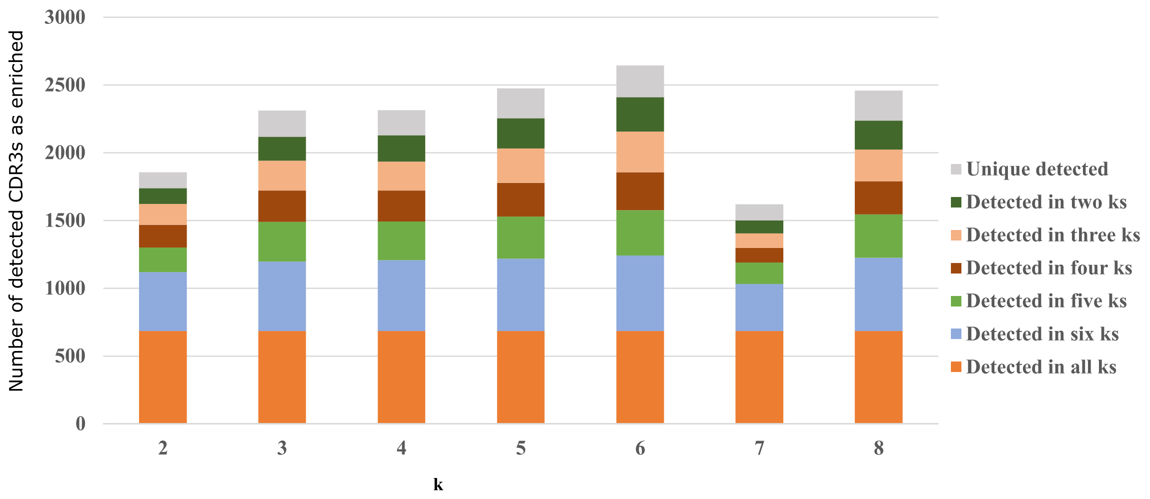

b)

k	# detected as Enriched	Known CD associated CDR3s	Running Time (hr)
2	1855	11	0.63
3	2311	11	1.02
4	2315	14	1.14
5	2475	12	1.80
6	2644	13	10.08
7	1619	8	18.20
8	2459	12	27.00

Figure 7S: Evaluation of the performance of nt k-mers with k 2 to 8. (a) A stacked bar plot of enriched CDR3s detected when using each k is shown. There is considerable overlap of the enriched CDR3s among the results of these ks, with those CDR3s detected by all ks constituting the highest overlap group (orange stack at the bottom), while the unique detected CDR3s making up the smallest proportion per each k (unique detected group). (b) For each k, the total number of CDR3s detected as enriched, the number of known CD associated CDR3s detected, and the running time taken are shown. The analyses for all ks was performed on CD PBMC dataset with 100 resample runs similar to the nt 4-mer analysis.

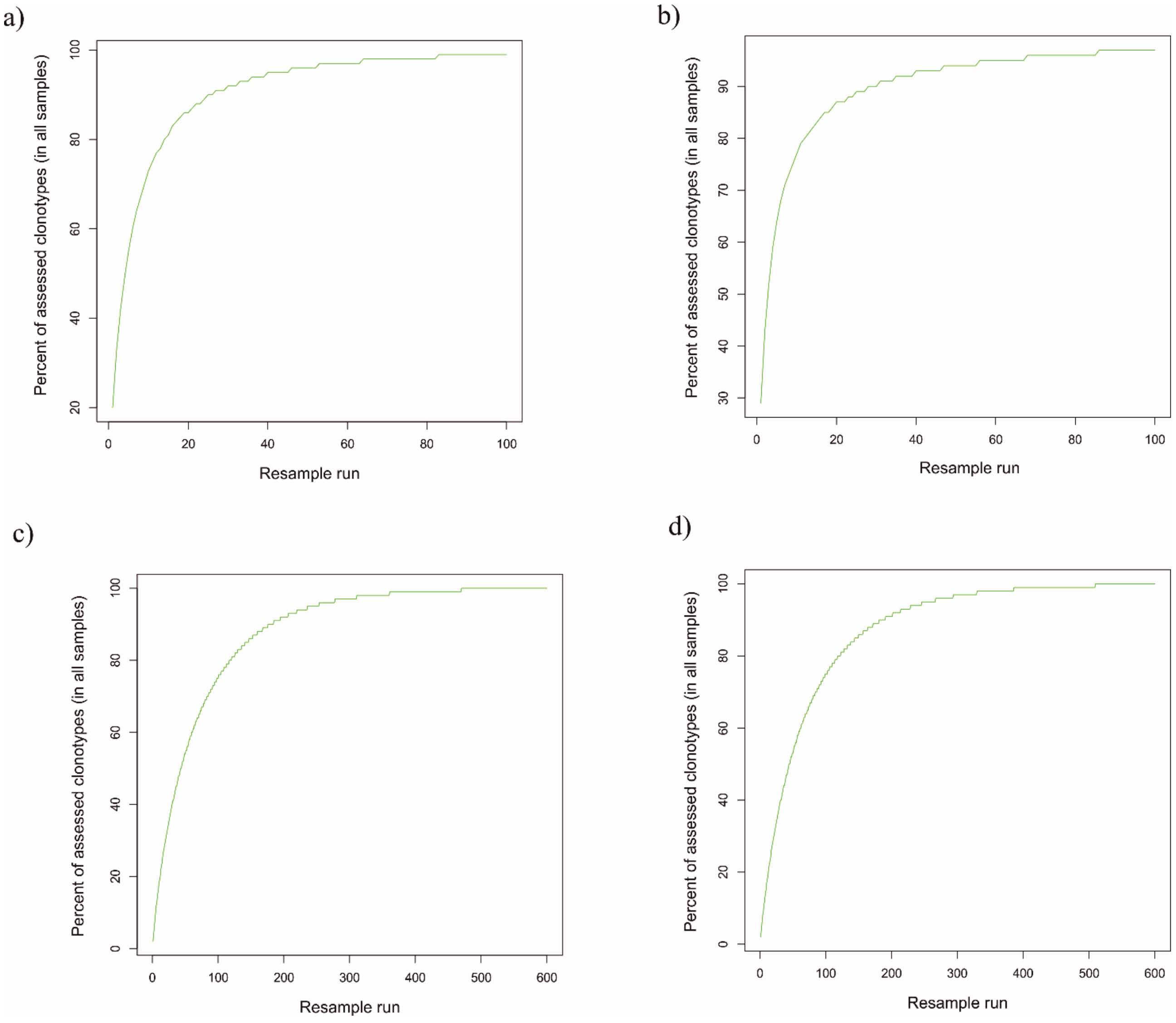

Figure 8S: Saturation plot shows nearly exhaustive assessment of all clonotypes the in each dataset. The number of downsample runs of steps 1 to 3 of the method is plotted against the proportion of new clonotypes assessed. Resample analysis rounds of 100 and 600 allow nearly 100% assessment of all clonotypes in CD PBMC (a), CD Gut (b), YFV PBMC (c), and twin YFV PBMC (d) datasets.

REFERENCES

1. Yohannes DA, Freitag TL, de Kauwe A, Kaukinen K, Kurppa K, Wacklin P, et al. Deep sequencing of blood and gut T-cell receptor β-chains reveals gluten-induced immune signatures in celiac disease. Sci Rep. 2017 Dec 21;7(1):17977.

2. DeWitt WS, Emerson RO, Lindau P, Vignali M, Snyder TM, Desmarais C, et al. Dynamics of the cytotoxic T cell response to a model of acute viral infection. J Virol. 2015 Apr;89(8):4517–26.

3. Robins HS, Campregher PV, Srivastava SK, Wacher A, Turtle CJ, Kahsai O, et al. Comprehensive assessment of T-cell receptor beta-chain diversity in alphabeta T cells. Blood. 2009 Nov 5;114(19):4099–107.

4. Lefranc M-P, Giudicelli V, Duroux P, Jabado-Michaloud J, Folch G, Aouinti S, et al. IMGT®, the international ImMunoGeneTics information system® 25 years on. Nucleic Acids Res. 2015 Jan;43(Database issue):D413-422.

5. Pogorelyy MV, Minervina AA, Touzel MP, Sycheva AL, Komech EA, Kovalenko EI, et al. Precise tracking of vaccine-responding T cell clones reveals convergent and personalized response in identical twins. Proc Natl Acad Sci. 2018 Dec 11;115(50):12704–9.

6. Bolotin DA, Poslavsky S, Mitrophanov I, Shugay M, Mamedov IZ, Putintseva EV, et al. MiXCR: software for comprehensive adaptive immunity profiling. Nat Methods. 2015 May;12(5):380–1.

7. Pogorelyy MV, Minervina AA, Chudakov DM, Mamedov IZ, Lebedev YB, Mora T, et al. Method for identification of condition-associated public antigen receptor sequences. eLife. 2018 Mar 13;7:e33050.

8. Pogorelyy MV, Minervina AA, Shugay M, Chudakov DM, Lebedev YB, Mora T, et al. Detecting T cell receptors involved in immune responses from single repertoire snapshots. Freeman TC, editor. PLOS Biol. 2019 Jun 13;17(6):e3000314.

9. Pogorelyy MV, Minervina AA, Shugay M, Chudakov DM, Lebedev YB, Mora T, et al. Detecting T-cell receptors involved in immune responses from single repertoire snapshots. bioRxiv. 2018 Jul 23;375162.

10. Qiao S-W, Ráki M, Gunnarsen KS, Løset G-\AAge, Lundin KE, Sandlie I, et al. Posttranslational modification of gluten shapes TCR usage in celiac disease. J Immunol. 2011;187(6):3064–3071.

11. Han A, Newell EW, Glanville J, Fernandez-Becker N, Khosla C, Chien Y-H, et al. Dietary gluten triggers concomitant activation of CD4+ and CD8+ αβ T cells and γδ T cells in celiac disease. Proc Natl Acad Sci U S A. 2013 Aug 6;110(32):13073–8.

12. Petersen J, Montserrat V, Mujico JR, Loh KL, Beringer DX, van Lummel M, et al. T-cell receptor recognition of HLA-DQ2-gliadin complexes associated with celiac disease. Nat Struct Mol Biol. 2014 May;21(5):480–8.

13. Nieweglowski L. clv: Cluster Validation Techniques [Internet]. 2013 [cited 2018 Nov 1]. Available from: https://CRAN.R-project.org/package=clv

14. Langfelder P, Zhang B, Horvath S. Defining clusters from a hierarchical cluster tree: the Dynamic Tree Cut package for R. Bioinforma Oxf Engl. 2008 Mar 1;24(5):719–20.
